# Proteasome inhibition targets the KMT2A transcriptional complex in acute lymphoblastic leukemia

**DOI:** 10.1038/s41467-023-36370-x

**Published:** 2023-02-13

**Authors:** Jennifer L. Kamens, Stephanie Nance, Cary Koss, Beisi Xu, Anitria Cotton, Jeannie W. Lam, Elizabeth A. R. Garfinkle, Pratima Nallagatla, Amelia M. R. Smith, Sharnise Mitchell, Jing Ma, Duane Currier, William C. Wright, Kanisha Kavdia, Vishwajeeth R. Pagala, Wonil Kim, LaShanale M. Wallace, Ji-Hoon Cho, Yiping Fan, Aman Seth, Nathaniel Twarog, John K. Choi, Esther A. Obeng, Mark E. Hatley, Monika L. Metzger, Hiroto Inaba, Sima Jeha, Jeffrey E. Rubnitz, Junmin Peng, Taosheng Chen, Anang A. Shelat, R. Kiplin Guy, Tanja A. Gruber

**Affiliations:** 1 Department of Pediatrics, Stanford University School of Medicine, Stanford, CA USA; 2 Department of Oncology, St. Jude Children’s Research Hospital, Memphis, TN USA; 3 Department of Computational Biology, St. Jude Children’s Research Hospital, Memphis, TN USA; 4 The University of Tennessee Health Science Center, Memphis, TN USA; 5 Department of Pathology, St. Jude Children’s Research Hospital, Memphis, TN USA; 6 Department of Chemical Biology and Therapeutics, St. Jude Children’s Research Hospital, Memphis, TN USA; 7 Center for Proteomics and Metabolomics, St. Jude Children’s Research Hospital, Memphis, TN USA; 8 Department of Pathology, University of Alabama School of Medicine, Birmingham, AL USA; 9 Department of Structural Biology, St. Jude Children’s Research Hospital, Memphis, TN USA; 10 Department of Developmental Neurobiology, St. Jude Children’s Research Hospital, Memphis, TN USA; 11 Department of Pharmaceutical Sciences, University of Kentucky, Lexington, KY USA; 12 Stanford Cancer Institute, Stanford University School of Medicine, Stanford, CA USA

**Keywords:** Acute lymphocytic leukaemia, Paediatric cancer

## Abstract

Rearrangments in Histone-lysine-N-methyltransferase 2A (KMT2Ar) are associated with pediatric, adult and therapy-induced acute leukemias. Infants with KMT2Ar acute lymphoblastic leukemia (ALL) have a poor prognosis with an event-free-survival of 38%. Herein we evaluate 1116 FDA approved compounds in primary KMT2Ar infant ALL specimens and identify a sensitivity to proteasome inhibition. Upon exposure to this class of agents, cells demonstrate a depletion of histone H2B monoubiquitination (H2Bub1) and histone H3 lysine 79 dimethylation (H3K79me2) at KMT2A target genes in addition to a downregulation of the KMT2A gene expression signature, providing evidence that it targets the KMT2A transcriptional complex and alters the epigenome. A cohort of relapsed/refractory KMT2Ar patients treated with this approach on a compassionate basis had an overall response rate of 90%. In conclusion, we report on a high throughput drug screen in primary pediatric leukemia specimens whose results translate into clinically meaningful responses. This innovative treatment approach is now being evaluated in a multi-institutional upfront trial for infants with newly diagnosed ALL.

## Abstract (web summary)

KMT2A rearranged infant acute lymphoblastic leukemia patients have a poor prognosis. Here, the authors use high throughput drug screening on primary infant specimens to identify a clinically active chemotherapy combination.

## Introduction

Acute lymphoblastic leukemia (ALL) in infants less than 1 year of age is a rare, but deadly disease. While pediatric ALL patients greater than 1 year of age enjoy a 5-year event-free-survival (EFS) of 85.6%, infants have a much poorer prognosis, with a 4-year EFS of only 47.7% on the recent Interfant06 study^1,2^. The poor outcomes seen in this subset of patients are believed to be a result of the unique biology of the leukemia cells, with approximately 80% of cases carrying a translocation that results in the rearrangement of *KMT2A* (KMT2Ar) and its fusion to one of more than 130 different partner genes^3^. Indeed, when limiting analysis to infants with a KMT2Ar, the 4-year EFS on Interfant06 only reached 38%^1^. We previously reported whole genome DNA sequence analysis on leukemic blasts and matched non-tumor tissue samples from 22 infant KMT2Ar cases and identified a mean of only 3.5 structural variations (SVs) and 2.2 somatic single nucleotide variations (SNVs) and/or insertions/deletions (indels) per case^4^. These data suggest that the major driver of transformation in this leukemia subtype is the translocation encoded KMT2A fusion protein, and thus efforts to improve outcomes with targeted therapeutics should focus on this genomic lesion. Despite the elucidation of DOT1L as a critical enzyme in the KMT2A transcriptional complex and the development of DOT1L inhibitors, objective response rates to this drug in adult phase I studies have been low^5–7^. While immunotherapy has been widely implemented in ALL, unique challenges in KMT2Ar infants provide barriers to these approaches including a propensity for lineage switch and low apheresis yields for CART production. Thus, there remains an urgent need for the development of alternative approaches to improve outcomes in these patients. To identify compounds that are active in an unbiased manner, we established a high throughput drug screening platform utilizing primary patient samples. Herein we identify a clinically active treatment combination and provide data to support a mechanism of action of proteasome inhibition in KMT2Ar leukemia whereby depletion of H2Bub1 leads to loss of the H3K79me2 mark imparted by the DOT1L enzyme. Consistent with impaired transcriptional elongation as a result of these epigenetic changes, KMT2A fails to sustain a presence at target genes triggering downregulation of the transcriptional program.

## Results

### Identification of active agents in KMT2Ar infant ALL using patient-derived xenograft cells

**Fig. 1:**
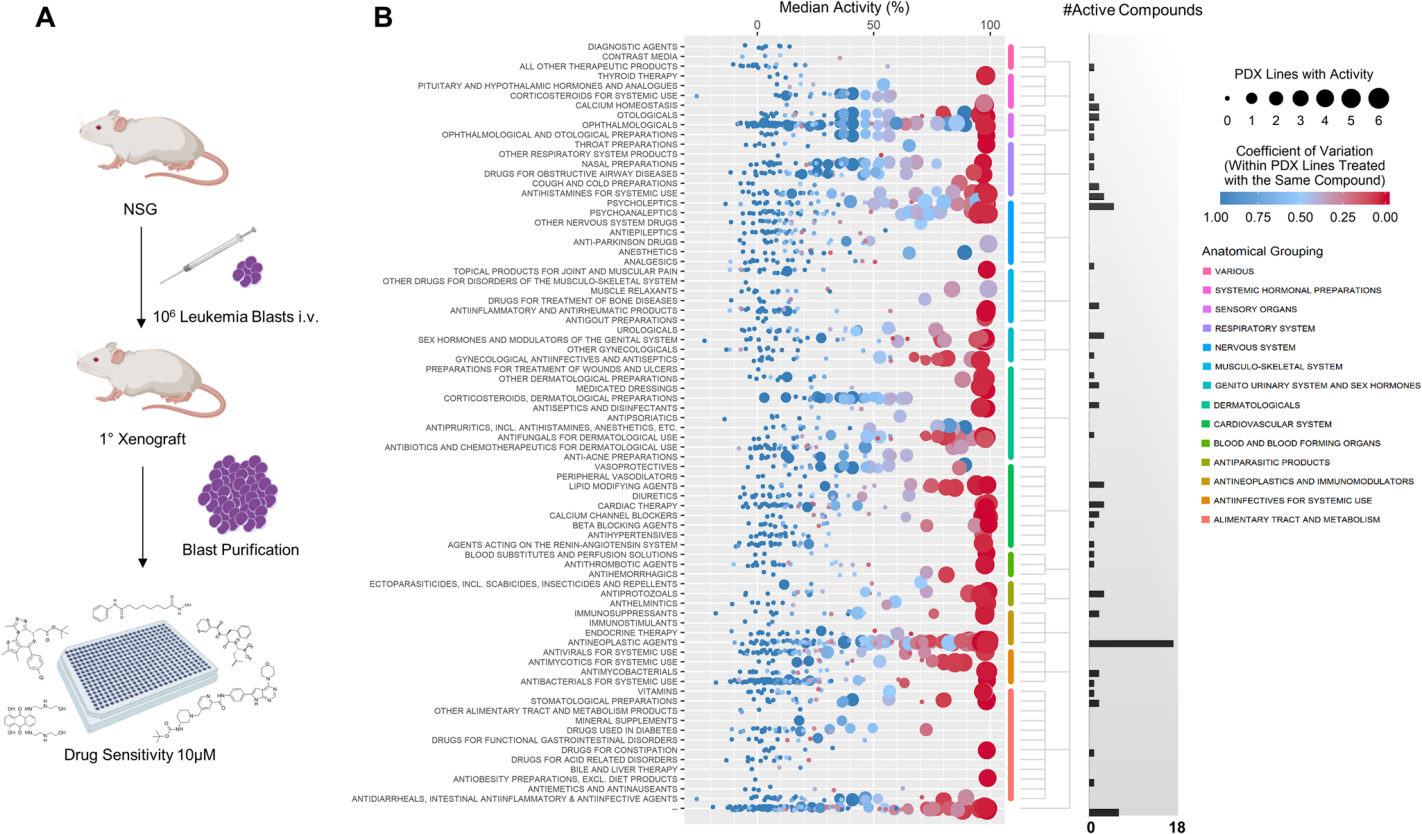
High throughput screening of infant ALL. **A** Study design. Primary leukemia blasts were thawed and injected into immunodeficient mice. Cells were harvested from moribund mice as described in “Methods” and viably stored in liquid nitrogen. Samples were thawed for drug sensitivity studies at a later time point. Figure created with BioRender.com, academic license. **B** In vitro activity of FDA-approved compounds. Six passaged patient specimens were thawed and cultured in the presence of compounds at a single concentration of 10 µM in duplicate as described in “Methods”. Normalized activity, defined as percent toxicity, was determined for each compound with each patient sample. Each data point corresponds to a single compound, the size of the data point indicates the number of patient samples with 80% or greater activity for that compound and the color indicates the coefficient of variation within the patient samples treated with that compound. Compounds are grouped according to class (see Supplementary Data 1). The number of compounds for each class with greater than 80% activity in five or more patient samples are shown. Source data are provided as a Source data file.

To identify compounds that are active in KMT2Ar leukemia, in vitro and in vivo assays were developed to evaluate drug sensitivity of primary ALL samples from infants (Fig. 1A). 15 primary human infant KMT2Ar leukemias were xenografted into NOD/SCID/IL2Rγnull (NSG) mice (Supplementary Table 1). All samples engrafted and expanded in NSG mice, leading to overt leukemia with a latency of 49 to 276 days (Supplementary Fig. 1A–C). Purification of leukemic blasts from a single moribund mouse yielded on average 10^8^ cells, providing sufficient material to screen large numbers of compounds. Culture of purified leukemic cells with cytokines on a collagen matrix supported growth in six of the patient specimens, allowing for a more accurate determination of drug sensitivity (Supplementary Fig. 1D). Growth in vitro correlated with early onset of disease in NSG xenografts as well as younger age at clinical presentation, allowing us to evaluate patient samples that represent aggressive high-risk disease (Supplementary Fig. 1E, F).

**Fig. 2:**
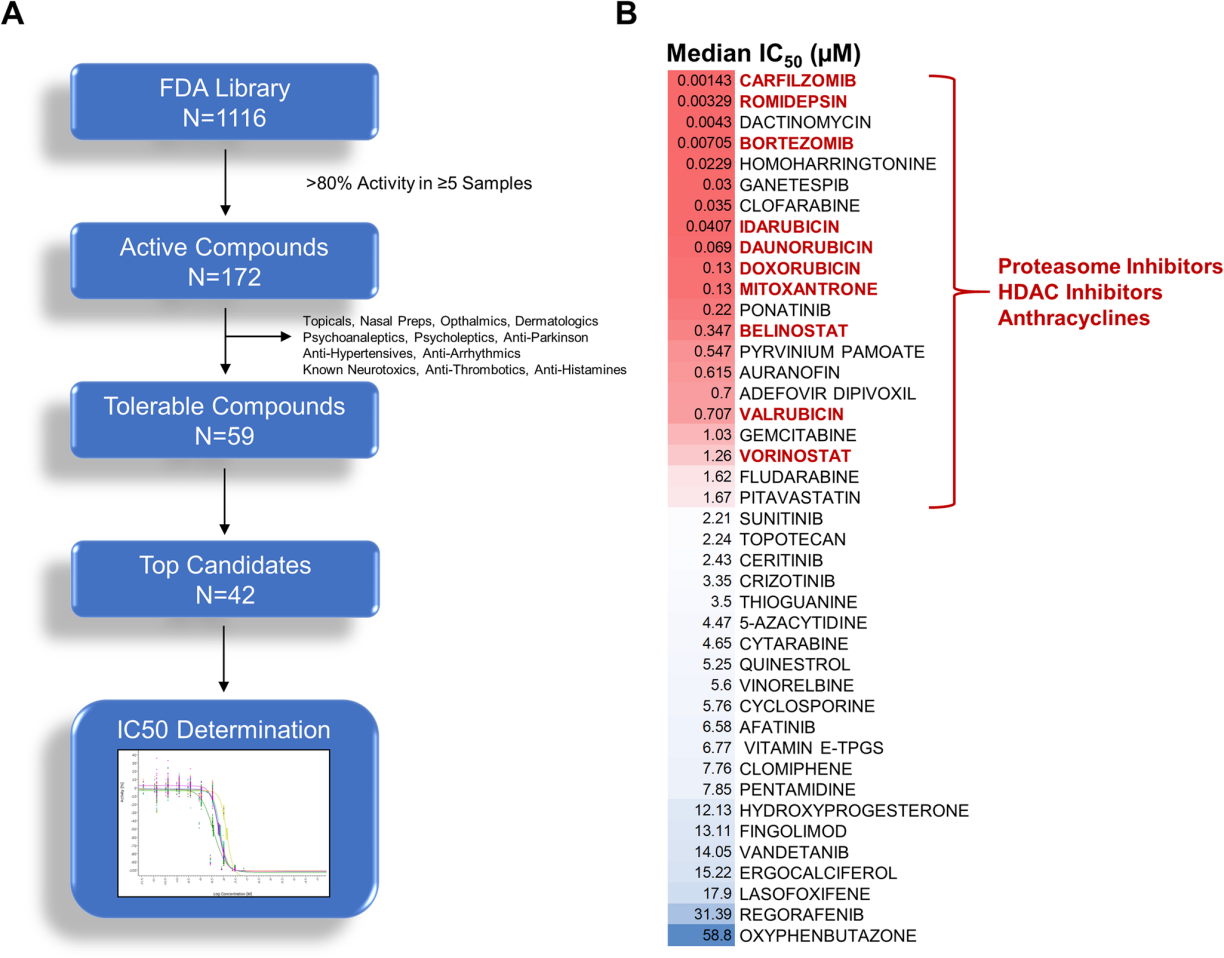
Active compounds. **A** Overview of FDA screening hits. 1116 unique compounds were screened. 172 compounds demonstrated >80% activity across five or more patient samples. Drugs with untolerable side effects or those that lacked systemic preparations were removed. The top 43 compounds underwent a secondary validation with a 10-point dose–response assay in technical triplicates. **B** Median IC_50_ values of top 43 compounds. 50% of the top compounds with IC_50_ values less than 2 µM belonged to one of three classes of agents: proteasome inhibitors, HDAC inhibitors, and anthracyclines.

The six patient specimens demonstrating growth in vitro underwent high throughput drug screening with an FDA-approved drug library of 1116 unique compounds at 10 µM (Fig. 1B, Supplementary Data 1). Viability was quantified by cellular ATP concentrations at 72 h which correlated with both metabolic activity and apoptosis as determined by the XTT assay and flow cytometry, respectively (Supplementary Fig. 2). 172 compounds demonstrated greater than 80% activity in at least five patient samples, with antineoplastic agents having the greatest number of active compounds (Figs. 1B, 2A, Supplementary Fig. 3, Supplementary Data 2). Following removal of compounds with non-systemic preparations or primary indications that would lead to undesirable side effects such as anti-hypertensives, 59 tolerable compounds were identified for further evaluation. The top 42 compounds were selected for secondary validation with 10-point dose–response assays to determine IC_50_ values (Fig. 2B, Supplementary Data 3, Supplementary Table 2). Three classes of agents compromised 50% of drugs that had the greatest activity with less than 2 µM IC_50_ values: anthracyclines, histone deacetylase inhibitors (HDACi), and proteasome inhibitors (Fig. 2B). Evaluation of additional non-FDA-approved HDACi and proteasome inhibitors confirmed the activity of these two classes of drugs with HDAC Class I/II pan inhibitors having greater potency overall compared to Class I or Class II specific inhibitors, with the exception of Romidepsin and OnKure’s OKI-5 (Supplementary Figs. 4, 5 and Supplementary Table 3). Expanding the analysis to include patient specimens that failed to grow in vitro also identified these three classes of drugs as having significant activity (Supplementary Table 2). In addition, specimens which failed to grow in vitro and represent lower risk disease as defined by age at presentation, were sensitive to standard ALL therapeutic agents such as antimetabolites and nucleoside analogs as previously reported (Supplementary Table 2)^8,9^. In contrast to anthracyclines and HDACi, where IC_50_ values were on par with those reported in the literature for primary childhood ALL samples, KMT2Ar infant samples required 10–100-fold less bortezomib, an FDA-approved proteasome inhibitor, to induce toxicity (Supplementary Table 2)^10^. Consistent with in vitro activity, treatment of INF001D engrafted NSG with bortezomib led to a significant reduction in disease burden as determined by spleen weight, although it failed to confer a survival benefit in this aggressive model (*p* = 0.033, Supplementary Fig. 6). Although proteasome inhibitors have broad activity in drug screens and are frequently a top hit, this differential response when contrasted with childhood ALL suggested a sensitivity with potential for clinical activity leading us to evaluate the compound further.

### Bortezomib depletes histone H2B ubiquitination leading to H3K79me2 depletion

**Fig. 3:**
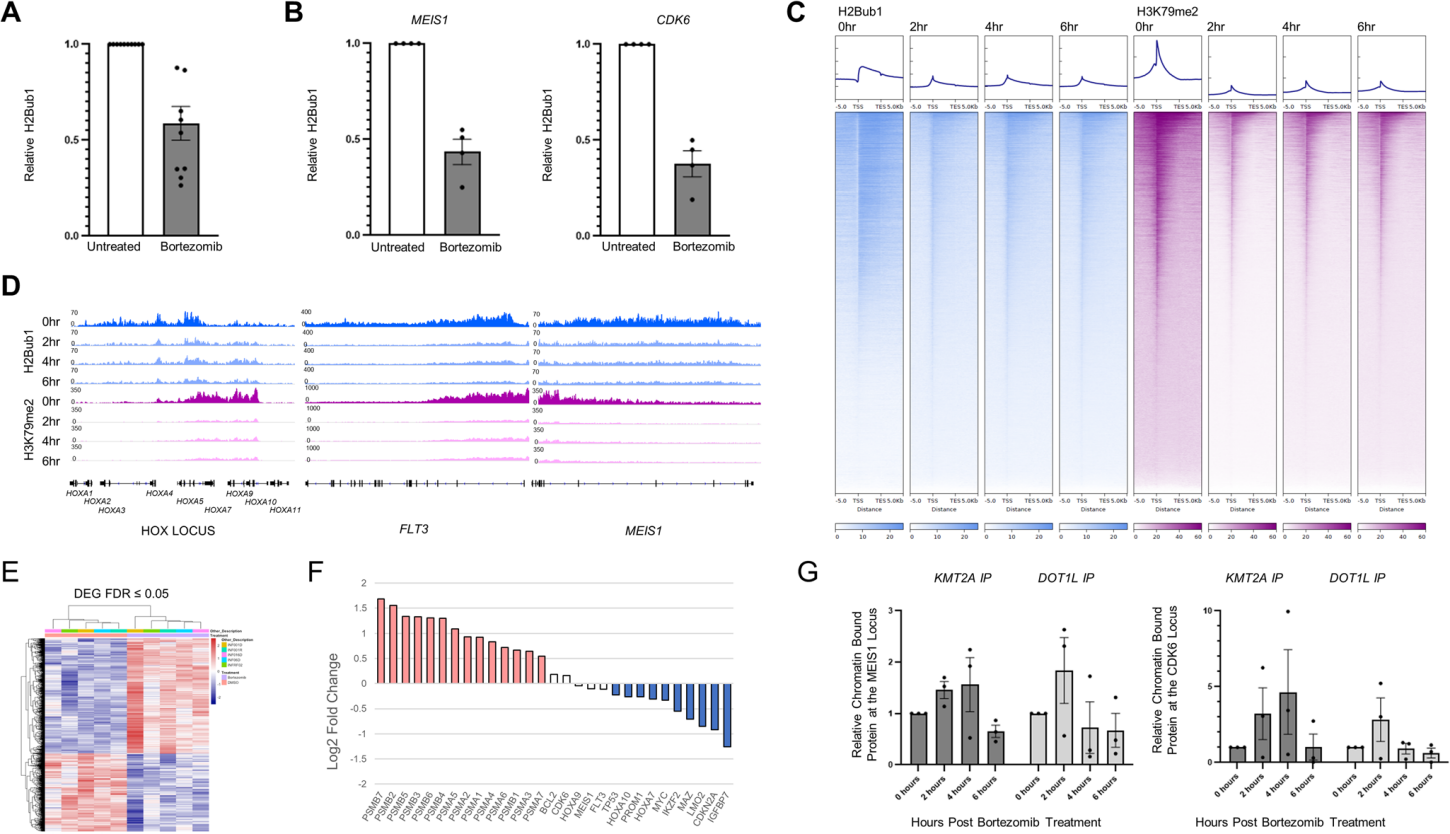
Bortezomib depletes H2Bub1 and H3K79me2 leading to downregulation of the KMT2A transcriptional program. **A** Ten patient samples were treated with 5 nM bortezomib, histones were extracted and blotted for total and ubiquitinated H2B. The ratio of H2Bub1 to total H2B levels was determined by densitometry. Pre-treatment specimens were set to one and relative levels at 2 h are shown with the standard error of the mean. **B** Four patient samples were exposed to 5 nM bortezomib for 6 h or left untreated. CUT&RUN reaction with an H2Bub1 antibody followed by quantitative PCR for two KMT2A target genes, *MEIS1* and *CDK6*, was done. Fold change over IgG was calculated and untreated specimens were set to one. The average across the four patient specimens with the standard error of the mean is shown. **C** ChIP-Rx of the KMT2Ar infant ALL cell line SEM at four time points (0, 2, 4, and 6 h following exposure to bortezomib) for H2Bub1 and H3K79me2 was done to quantitatively assess this epigenetic mark over time throughout the genome. Heatmaps of H2Bub1 and H3K79me2 genome-wide are shown. **D** Profiles at *HOXA1-A11*, *FLT3*, and *MEIS1* gene loci. **E** Five patient specimens were grown in the presence or absence of 5 nM bortezomib for 20 h followed by RNA extraction from 2 million live cells and sequencing. Cells were analyzed at 20 h based on the half-life of mRNA which is 10 h. A heatmap of differentially expressed genes is shown. **F** Log2 fold change of proteasome complex transcripts, KMT2Ar target genes, and the cMYC target genes TP53 and CDKN2A are shown following exposure to bortezomib. The upregulation of proteasome complex genes demonstrates target inhibition. **G** The KMT2Ar infant ALL cell line (SEM) was grown in the presence or absence of bortezomib. CUT&RUN reactions at 0, 2, 4, and 6 h following exposure to bortezomib was done with an N-Terminal KMT2A antibody and DOT1L. Quantitative PCR for two KMT2A target genes, *MEIS1* and *CDK6* was determined. Fold change over IgG was calculated and untreated specimens were set to one. The average across four independent experiments are shown with the standard error of the mean. Source data are provided as a Source data file.

Bortezomib has been shown to mediate responses through several mechanisms, including NFkB inhibition, stabilization of cell cycle regulatory proteins, and induction of apoptosis^11^. Proteasome inhibition has been demonstrated to lead to accumulated KMT2A fusion protein levels, triggering apoptosis and cell cycle arrest in KMT2Ar cell lines providing one mechanism for the effectiveness seen in our system^12^. To determine if NFkB inhibition also plays a role in sensitivity of KMT2Ar to bortezomib, we evaluated cellular concentrations of the activated NFkB transcription factor but failed to see decreased levels upon treatment of cells with this agent (Supplementary Fig. 7). Bortezomib has also been shown to deregulate ubiquitin stores and deplete histone H2B ubiquitination (H2Bub1), an epigenetic mark that is linked to histone methylation and expression^13–15^. Several groups have published reports demonstrating H2Bub1 is required for DOT1L activity, the enzyme responsible for histone methylation and establishment of the KMT2A gene expression program^16–18^. We therefore evaluated H2Bub1 levels in KMT2Ar cell lines and patient samples in the presence of bortezomib and confirmed rapid depletion of this epigenetic mark by western blotting of histone extracts (*N* = 10 patient samples) and CUT&RUN qPCR (*N* = 4 patient samples) at two KMT2Ar target genes: *MEIS1* and *CDK6* (Fig. 3A, B, Supplementary Fig. 8). The depletion of H2Bub1 was also seen in response to carfilzomib exposure but not other chemotherapeutic drugs including cytarabine, vincristine, and dexamethasone, consistent with a proteasome inhibitor class effect (Supplementary Fig. 9). Furthermore, quantitative genome-wide chromatin immunoprecipitation sequencing (ChIP-Rx) of bortezomib-treated KMT2Ar SEM cells demonstrated a concordant decrease in histone 3 methylation at lysine residue 79 (H3K79me2) both globally and at KMT2A-AFF1 target genes, the epigenetic mark imparted by DOT1L, within two hours (Fig. 3C, D, Supplementary Fig. 10, Supplementary Data 4). Consistent with these data, patient samples treated with bortezomib downregulated KMT2A target genes such as *HOXA10*, *PROM1*, *MYC*, *LMO2*, *IGFBP7* as well as downstream cMYC target genes *TP53* and *CDKN2A* at 20 h (Fig. 3E, F, Supplementary Data 5), demonstrating that the KMT2A transcriptional program is inhibited in the presence of bortezomib. Although 20 h allowed for two mRNA half-life to occur, poor viability at later time points prevented us from extending the incubation further when residual transcripts would be completely eliminated. Accumulation of full-length KMT2A from the non-rearranged allele in bortezomib-treated cells was limited to the cytosolic fraction arguing against downregulation of the KMT2A oncogenic transcriptional program via competition for components of the transcriptional complex by wild-type KMT2A (Supplementary Fig. 11). CUT&RUN qPCR of two KMT2A-AFF1 target genes for KMT2A and DOT1L revealed an initial increase in binding at 2–4 h which dropped precipitously by 6 h, suggesting a failure for both the wild type and fusion complexes to sustain a presence at target genes (Fig. 3G). To confirm this finding, we conducted CUT&RUN sequencing of KMT2A using an N-terminal antibody that recognizes both the fusion and wild-type KMT2A which revealed a decrease in binding genome-wide and at target loci (Supplementary Fig. 12). We conclude that in addition to previously published mechanisms of sensitivity to proteasome inhibition in KMT2Ar leukemia, the depletion of H2Bub1 impairs expression of the KMT2Ar transcriptional program contributing to the activity of this class of agents in this aggressive leukemia subtype.

### Bortezomib and histone deacetylase inhibitors in combination are highly active against KMT2A rearranged leukemia

**Fig. 4:**
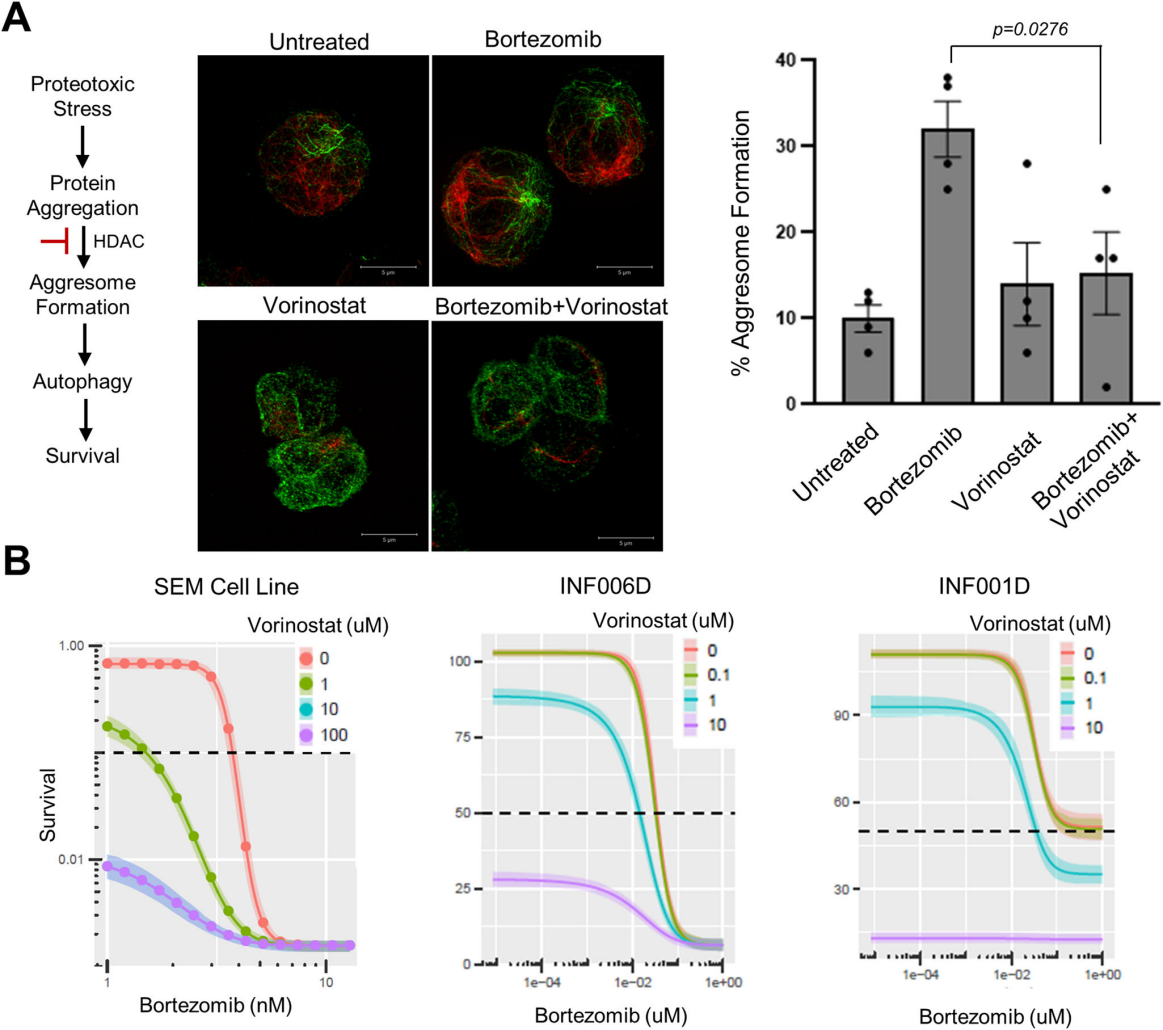
Vorinostat is synergistic with bortezomib in KMT2Ar leukemia. **A** SEM cells were incubated for 8 h in 3 nM bortezomib, 1 µM vorinostat, or both followed by staining for acetylated tubulin (green) and vimentin (red) to visualize microtubules and aggresomes, respectively. Representative images are shown. 100 cells were scored per group by three individuals independently for the presence of aggresomes, average counts with the standard error of the mean are shown. Unpaired t test comparing bortezomib to bortezomib+vorinostat, two-tailed *p* value equals 0.0276. Source data are provided as a Source data file. **B** Cytotoxicity assay of bortezomib in combination with vorinostat. The KMT2Ar infant ALL cell line SEM and two patient samples were grown in the presence of bortezomib and vorinostat at the indicated concentrations for 72 h in technical triplicates. Viability was assessed by cell titer glo. Estimated effect of various levels of vorinostat in combination with bortezomib are shown. Curve shifts to the left indicate potentiation. EC50 values of bortezomib in the presence of varying concentrations of vorinostat are presented in Table 1. See Supplementary Fig. 17 for the best fit BRAID surface for each combination with estimated value of κ and confidence interval.

Similar to proteasome inhibitors, HDACi have a broad mechanism of action and are often identified as having in vitro activity across multiple tumor types. We noted however that vorinostat has been shown to be synergistic with bortezomib in multiple models and has been demonstrated to have single-agent activity in KMT2A rearranged leukemia, both in our study and others (Fig. 2B, Supplementary Figs. 3, 4, Supplementary Table 3)^19–24^. We reasoned therefore that a combinatorial approach may provide benefit. The basis of synergy between proteasome and HDAC inhibition is multifactorial; one mechanism is the dual inhibition of the proteasome and aggresome pathways by bortezomib and HDACi, respectively, leading to a greater accumulation of polyubiquitinated proteins, resulting in increased cellular stress and apoptosis^25,26^. In addition, histone hyperacetylation which promotes death gene induction is reported to be potentiated when cells are exposed to bortezomib in combination with HDACi suggesting that the synergy is reciprocal between the two classes of agents^21,27^. To evaluate these possibilities, we first treated KMT2Ar cells with bortezomib followed by a multiplexed mass spectrometry-based proteomic analysis at multiple time points over 20 h to further understand the cellular response of these cells to proteasome inhibition^28^. 20 h was chosen as cells were demonstrating signs of apoptosis at this time point as indicated by trypan blue exclusion (data not shown). 9514 proteins were quantified in this analysis of which 3837 were differentially expressed over 20 h by ANOVA (*p* < 0.01, Supplementary Fig. 13, Supplementary Data 6, 7). Differential enrichment analysis using limma identified the P53, G2M Checkpoint, and Apoptosis pathways to be positively enriched at 0, 6, 12, and 16 h confirming the cellular stress imparted by bortezomib (Supplementary Fig. 14 and Supplementary Data 8–20)^29^. By 20 h, downregulation of Glycolysis was found consistent with apoptotic cells (Supplementary Data 12). Consistent with prior reports, we saw an accumulation of several KMT2A transcriptional complex proteins in total protein extracts as well as evidence of the unfolded protein response and ER stress (Supplementary Fig. 15). Evaluation of histone acetyltransferases (HATs) and HDAC proteins revealed a reduction in several Class I, IIb and III HDACs upon treatment with bortezomib as well as an increase in two HATs (supplementary Fig. 16, Supplementary Table 4). In agreement with this data histone acetylation increased upon exposure to single-agent bortezomib (Supplementary Fig. 16). Combinatorial treatment with vorinostat and bortezomib, however, did not further enhance histone acetylation beyond treatment with vorinostat alone (Supplementary Fig. 16). We therefore assessed aggresome formation by immunofluorescence of vimentin in response to single-agent bortezomib and found that it was significantly inhibited in the presence of vorinostat providing one mechanism of potential synergy in our system (Fig. 4A). To evaluate enhanced cytotoxicity with combinatorial treatment we subjected the KMT2Ar infant ALL cell line SEM and patient samples to treatment with both bortezomib and vorinostat in a dose–response assay and found that vorinostat potentiated the effects of bortezomib leading to greater cell kill with lower concentrations of bortezomib, in agreement with the inhibition of aggresome formation seen (Fig. 4B, Table 1). An analysis of fitted response surfaces was consistent with an additive effect when the drugs were used in combination (Supplementary Fig. 17)^30^. Further, the addition of vorinostat to bortezomib in the treatment of INF001D engrafted NSG led to a statistically significant prolonged survival compared to single-agent bortezomib (*p* = 0.005, Supplementary Fig. 6). We conclude that vorinostat and bortezomib are a highly active combination in KMT2Ar leukemia through multiple mechanisms.

**Table 1:** Vorinostat and bortezomib combinatorial treatment

Vorinostat concentration	EC50 bortezomib		
	SEM (mean, range)	INF006D (mean, range)	INF001D (mean, range)
0 µM	3.74 nM (3.55–3.92 nM)	34.9 nM (31.4–37.6 nM)	Inf (108–Inf nM)
0.1 µM	ND	32.4 nM (30.2–34.3 nM)	Inf (125–Inf nM)
1 µM	1.5 nM (1.35–1.62 nM)	14.4 nM (12.1–16.7 nM)	35.3 nM (29.1–44.1 nM)
10 µM	0 nM (0–0 nM)	0 nM (0–0 nM)	0 nM (0–0 nM)

*Inf* infinity, *ND* not determined.

### Bortezomib, vorinostat, and mitoxantrone combination therapy induce high response rates in relapsed refractory disease

**Fig. 5:**
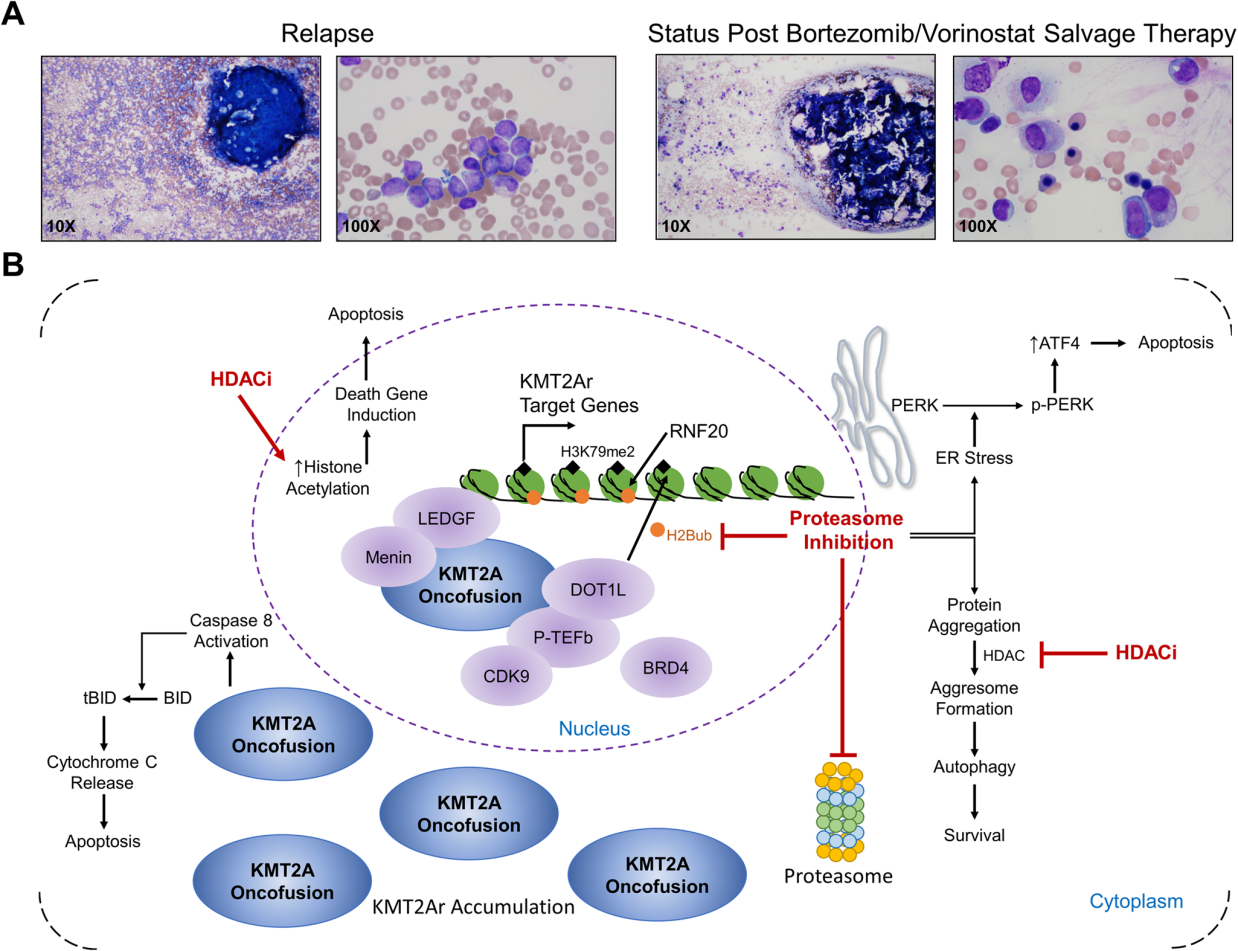
Restoration of trilineage hematopoiesis following treatment with bortezomib and vorinostat containing chemotherapy regimen in a refractory patient. **A** Bone marrow aspirates of case 3b (see Table 2) at the time of relapse post-transplant and post-treatment with bortezomib and vorinostat-containing chemotherapy regimen are shown at ×10 and ×100 magnification. Flow-based MRD and RT-PCR for the *KMT2A-MLLT1* oncogene present in this patient were both negative post-treatment. **B** Model of proteasome and HDAC inhibition in KMT2Ar leukemia. Proteasome inhibitors deplete H2Bub1 leading to downregulation of the KMT2A gene expression program and accumulation of the KMT2A fusion which triggers caspase 8^12^. HDACi prevent aggresome formation and increase in histone acetylation and death gene induction promoting apoptosis.

Based on encouraging data of bortezomib in Phase I and II pediatric ALL trials, three relapsed/refractory infants that failed multiple salvage attempts received treatment with bortezomib in combination with a four-drug anthracycline-containing chemotherapy backbone as previously published (Supplementary Tables 5, 6)^31–33^. Surprisingly, bortezomib demonstrated significant activity with two patients achieving morphologic remission as defined by less than 5% blasts (Table 2 Cases 1–3a, Supplementary Table 5 Cases 1–3a). All three relapsed/refractory infants were subsequently transplanted despite the presence of minimal residual disease (MRD) with either haploidentical or match unrelated donor transplants. While the first two patients died of treatment-related mortality, patient 3 remained in remission for 210 days post-transplant but subsequently relapsed. At that time, the patient was re-induced with the bortezomib-containing multi-agent chemotherapy regimen with the addition of vorinostat (Table 2 Case 3b, Fig. 5A, Supplementary Table 5 Case 3b). With this single course the patient achieved MRD negative status by flow cytometry following count recovery, a response greater than that achieved by the addition of bortezomib alone to the chemotherapy backbone prior to transplant (Table 2 Case 3b, Supplementary Table 5 Case 3b). Four relapsed/refractory KMT2Ar acute myelogenous leukemia (AML) patients also received salvage regimens with the combination of bortezomib, vorinostat, and mitoxantrone, all of whom responded (Table 2 Cases 5–8, Supplementary Table 5 Cases 5–8). The most frequent adverse events were infectious in nature with six of the ten patients experiencing one or more grade 3–4 infections (Supplementary Table 6). Two patients had a grade 4 event: a pseudomonas sepsis (case 3b) and a pre-existing adenoviral infection that advanced from grade 3 to grade 4 (case 6) (Supplementary Table 6). This suggests that the activity of these three classes of drugs, identified in our laboratory assays, are active against all KMT2Ar leukemias and not restricted to the lymphoid subset of patients. The achievement of MRD negative status was not attained until vorinostat was included, confirming the additive effect seen in our laboratory assays. Overall, for KMT2Ar patients receiving bortezomib, vorinostat, and mitoxantrone, we saw a complete response rate (CR + CRi) of 80% (8/10 patients; Table 2) and an overall response rate of 90% (9/10 patients; Table 2). This compares very favorably with recent relapsed ALL and AML trials that have reported CR rates between 40–65% using the same criteria of <5% leukemic blasts^31,32,34^.

**Table 2:** Clinical responses to bortezomib and vorinostat containing salvage regimens

Case	Phenotype^a^	Chemo doses		Pre-treament		Post-treatment		
		Bortezomib^b^	Vorinostat^b^	%Blast^c^	%MRD^c^	%Blast^c^	%MRD^c^	Response^d^
1	ALL	4	0	75	94.9	1	3.23	CRi
2	ALL	4	0	>90	ND	12	7.93	PRi
3a^e^	ALL	6	0	83	86	0	1.38	CR
3b^e^	ALL	8	12	83	ND	2	<0.01	CRi
4	ALL	6	12	74	67	60	48.14	SD
5	AML	8	12	52	68	0	2	CR
6^f^	AML	8	12	46	23	26	<0.01	CRi
7	AML	6	12	8	5.87	2	<0.01	CR
8	AML	8	16	8	12.8	0	0.28	CRi
9	MPAL	6	12	86	78	0	0.997	CRi
10	ALL	8	16	46	30.8	0	<0.01	CR

*MPAL* mixed phenotype acute leukemia.

^a^Immunophenotype by flow cytometry.

^b^Total number of doses of bortezomib and vorinostat given over the course of the treatment. All patients received two doses of mitoxantrone and additional chemo as described in Supplementary Table 5.

^c^Blast percentage as determined by morphology; MRD percentage as determined by flow cytometry.

^d^CR (complete response), CRi (complete response with incomplete blood count recovery), PRi (partial response with incomplete blood count recovery), SD (stable disease).

^e^Case 3a course was pre-transplant, case 3b is the same patient course given post-transplant.

^f^Case 6 had a systemic adenoviral infection prior to and post chemotherapy. At the time of evaluation post treatment, while morphologic blasts were elevated, this was felt to be a recovering marrow given negative MRD result by flow and PCR.

## Discussion

As outcomes for pediatric ALL continue to improve over time across multiple cooperative group studies, the lack of progress for infants with this disease, despite a large body of scientific literature delineating the mechanism of KMT2Ar leukemogenesis, has proven frustrating for clinicians and scientists alike. In contrast to childhood ALL, infants with KMT2Ar ALL fail to respond to standard of care chemotherapeutic agents necessitating trial of novel agents. This poses a conflict with the current system of drug development that initiates in adults and trickles down to pediatrics. Even within the field of pediatrics, many clinicians are hesitant to investigate novel agents in our youngest and arguably most vulnerable patients. Compounded by a lack of suspension formulations for many new drugs as well as the rarity of the diagnosis, trial development for KMT2Ar infants remains a challenge. We sought to establish a platform in which promising agents could be defined, prioritized and studied in the laboratory, providing a source for evaluation in the clinical setting. As proof of principle, we evaluated 1116 FDA-approved compounds to determine the applicability and robustness of our system. Consistent with historical clinical experience, standard ALL drugs, with the exception of anthracyclines, failed to show significant activity in patient samples with high-risk features. In contrast, two newer classes of agents, HDAC and proteasome inhibitors, were able to induce apoptosis at clinically achievable concentrations. Furthermore, in a small number of relapsed/refractory patients, salvage chemotherapy regimens including these drugs resulted in strong response rates. Since our preliminary findings were reported at the American Society of Hematology, another group has confirmed the preclinical activity of proteasome inhibitors with studies on carfilzomib in infant ALL cell lines^35,36^. Similar to our findings, single-agent treatment of PDX models with carfilzomib failed to confer a survival benefit, nor was a survival advantage seen when given in combination with vincristine, dexamethasone, and L-asparaginase. Our data suggest that the addition of an HDAC inhibitor plays an important role in sustaining a response.

The sensitivity of our patient specimens to the proteasome inhibitor bortezomib led us to investigate if there was an additional mechanism whereby KMT2Ar leukemia cells were susceptible to this drug. Studies demonstrating a role for histone ubiquitination in the KMT2A gene expression program, in addition to the known effect of proteasome inhibition on this mark, led us to consider the possibility that bortezomib altered the epigenetic landscape of the leukemia cells. Consistent with this hypothesis, we found treatment of cells with bortezomib led to a depletion of H2Bub1 and a genome-wide decrease in H3K79me2, the modification imparted by the DOT1L enzyme that associates with KMT2A fusion proteins to activate transcription. This contributes further to the large amount of data linking the proteasome with epigenetics^37^. Given the role of H2Bub1 in gene regulation, further studies to delineate epigenetic alterations from proteasome inhibition in other malignancies should yield additional mechanisms of action beyond that which are currently known for this class of drugs.

We propose that treatment with bortezomib results in a depletion of H2Bub1 leading to an indirect inhibition of DOT1L (Fig. 5B). Yet direct inhibition of DOT1L has failed to achieve durable responses in phase I clinical trials^7^. In contrast to DOT1L inhibitors which are targeted, bortezomib has pleomorphic effects on cancer cells that likely also contribute to the activity seen in our studies. In agreement with this, proteasome inhibition has been shown to trigger apoptosis through activation cleavage of BID by Caspase-8 in KMT2Ar ALL cells^12^. In addition, bortezomib has significant activity in relapsed childhood ALL consistent with non-KMT2A dependent mechanisms of action^32,33^. Importantly, treatment of patients with leukemia requires multi-agent therapy. The belief in this concept combined with the strong desire to cure all of our patients, has caused a shift in many pediatric relapsed studies from single-agent trials to those evaluating the activity of novel agent(s) in combination with so-called “standard” chemotherapy drugs^38–40^. Bortezomib has been demonstrated to be additive or synergistic with multiple drugs, including steroids, HDACi, vincristine, and others^24,41^. The promising responses in our relapsed/refractory patients occurred with chemotherapy regimens including several drugs known to potentiate the effects of bortezomib. To determine if this approach translates into improved outcomes, the combination of bortezomib and vorinostat in a chemotherapy backbone for newly diagnosed infant ALL patients is now being formally evaluated in a multi-institutional trial (ClinicalTrials.gov Identifier: NCT02553460). End of induction MRD responses for patients enrolled thus far have greatly exceeded historical rates with 72% of patients carrying KMT2Ar achieving MRD negative status three weeks into therapy (*N* = 36, Gruber et al., unpublished data)^42^. Whether these early robust responses will translate into a survival advantage has yet to be determined.

In summary, we have established a platform for screening compounds on primary leukemia specimens and demonstrate its clinical utility using an FDA-approved library. This approach can further be broadened to evaluate larger chemical libraries to identify as yet unknown cellular vulnerabilities and potential therapeutic targets.

## Methods

This research complies with all relevant ethical regulations; patient samples and treatment were approved by the St. Jude Children’s Research Hospital Institutional Review Board and animal studies were approved by the St. Jude and Stanford Institutional Animal Care and Use Committees.

### Patient-derived xenografts

Patient samples were obtained from St. Jude Children’s Research Hospital with parent/guardian consent under protocols approved by the Institutional Review Board and stored in liquid nitrogen until use. Experiments involving mice were reviewed and approved by the Institutional Animal Care and Use Committee at St. Jude and Stanford. Female NSG mice purchased from Jackson Laboratories, age 4 to 8 weeks, were irradiated with 250 rads one day prior to transplant. Mice were housed with a 12 h light/12 h dark cycle with temperatures between 65–75 °F and 40–60% humidity. 10 mice per patient sample were transplanted with 1 × 10e^6^ leukemia cells intravenously by tail vein injection. Moribund mice were sacrificed and patient cells were purified as described in the following section. For murine drug treatment trials, NSG mice, age 4–8 weeks were irradiated with 250 rads one day prior to transplant. Mice were transplanted with 1 × 10e^6^ leukemia cells from INF001D on day 0. Engraftment was verified on day 28 by flow cytometry of the peripheral blood. Control mice received PBS injections, while bortezomib-treated mice received 0.1 mg/kg/dose intravenously (BTZ) twice weekly beginning on day 32. Vorinostat (V) was administered by oral gavage once daily at 100 mg/kg/dose on days 32–35, 39–42, 46–49, and 53–56. Please see Supplementary Fig. 6 for engraftment and treatment schema. Clinical signs of illness include decreased activity, weight loss, decreased grooming, hind limb paralysis, and hunched posture. Mice demonstrating any clinical signs of illness and/or 15% weight loss were euthanized according to institutional guidelines.

### Purifying xenograft samples

Peripheral blood, bone marrow, and spleen were collected from moribund mice. Peripheral blood was stained with hCD3 FITC (BD 349201), hCD19 PE (BD 340720), hCD45 PeCy7 (BD 557748), and mCD45 APC (BD 553991) and analyzed using FACS LSR II BD (BD Biosciences). Upon sacrifice, xenografted cells from the bone marrow and spleen were separated from murine cells by staining with hCD45 microbeads followed by negative selection of murine cells with LS columns according to manufacturer’s protocol (MACS Miltenyi Biotec), and frozen for subsequent drug sensitivity studies. For a given patient sample, cells from each mouse were pooled for screening.

### High throughput drug screen

Samples were thawed and plated at a density of 5625 cells per well in collagen-coated 384-well cell culture plates (BD BioCoat). Cells were plated in triplicate in 25 µl of StemPro®−34 SFM medium (Thermofisher) supplemented with MEM NEAA (Gibco), Pen-step (Gibco), L-Glutamine (600 mM Invitrogen), hIL3 (20 ng/ml PeproTech), hIL7 (10 ng/ml PeproTech), and hFLT3 (20 ng/ml PeproTech). Plates were incubated overnight in a humidified 37 °C incubator with 5% CO_2_. Drugs were added to a 384-well plate the following morning after being validated and checked for purity. The drugs and concentrations used on plate can be found in Supplementary Data 1. All compounds were prepared in DMSO and are greater than 95% pure except for Vincristine (80–85% pure), as well as Vinorelbine and MLN 4924 whose purity could not be determined. High throughput screening was performed with an automated screening system (HighRes Biosolutions). Drugs were transferred with a pin tool (V&P Scientific) in amounts ranging from 67 to 73 nl. Plates were incubated at 37 °C, 95%rH, and 5% CO_2_ for 72 h following the addition of drugs. After equilibrating the plates to room temperature, cell number was determined by adding 25 µl of Cell Titer Glo Reagent (Promega) to each well and luminescence was read on an Envision plate reader (Perkin Elmer). Dose–response curves and EC_50_ values were calculated using Genedata Screener v15 (Genedata). Aggregation of compound activities across xenografts was performed in R (https://cran.r-project.org/) and plotted with the ggplot2 v3.4.0 package using annotations from the World Health Organization Anatomical Therapeutic Chemical Classification^43^. Activity values in Supplementary Data 2 are the normalized individual activities for a given compound in a given sample as determined by luminescence. Negative controls (DMSO treated) are centered around 0 and positive controls are centered around −100, therefore the activity values are negative and a value of −99 corresponds to 99% killing.

### XTT assay

Exponentially growing cells were plated 30,000 cells per well in 96-well collagen-coated plates (BD BioCoat) in 100 µl of media. Media used to plate the cells included 50x serum replacement (Sigma), MEM NEAA (Gibco), Pen-step (600 mM Gibco), L-Glutamine (Invitrogen), hIL3 (20 ng/ml PeproTech), hIL7 (10 ng/ml PeproTech), hFLT3 (20 ng/ml PeproTech), and Stem Pro-34 SFM with nutrient supplement (Invitrogen). Plates were incubated overnight in a humidified 37 °C incubator with 5% CO_2_. Prednisolone, Vincristine, Bortezomib, and Mitoxantrone were then manually added to cells in concentrations ranging from 10 to 0.005 µM and plates were then placed in the incubator for an incubation period of 72 h. XTT labeling reagent and electron-coupling reagent from Cell proliferation Kit II (Roche) were added to cells as per manufacturer’s instructions and incubated for 24 h. Absorbance was then read on a Synergy microplate reader (Biotek).

### Apoptosis assay

Exponentially growing cells were plated 178,000 cells per well in 24-well collagen-coated plates (BD BioCoat) in 500 µl of media. Media used to plate the cells included 50x serum replacement (Sigma), MEM NEAA (Gibco), Pen-step (Gibco), L-Glutamine (600 mM Invitrogen), hIL3 (20 ng/ml PeproTech), hIL7 (10 ng/ml PeproTech), hFLT3 (20 ng/ml PeproTech), and Stem Pro-34 SFM with nutrient supplement (Invitrogen). Plates were incubated overnight in a humidified 37 °C incubator with 5% CO_2_ Prednisolone, Vincristine, Bortezomib, and Mitoxantrone were then manually added to cells in concentrations ranging from 10 to 0.005 µM and plates were then placed back in the incubator for an incubation period of 72 h. Cells were removed from each plate, and suspended in PBS, stained with AnnexinV FITC and 7-AAD (BD Biosciences), and analyzed within one hour using FACS LSR II BD (BD Biosciences).

### NFkB ELISA

To quantitate active NFkB, cell lines and patient samples were treated with 50 nM and 5 nM of Bortezomib, respectively, for 2 h. Nuclear NF-kB p50/p65 binding activity was measured by ELISA as per manufacturer’s instructions (Enzo Life Sciences cat# ADI-EKS-446). As a control the murine fibroblast cell line NIH3T3 was treated with 20 ng/ml recombinant TNFα for ten minutes.

### IKBα western blot

To evaluate IKBα and phosphorylated IKBα levels, total protein was extracted from cells using RIPA lysis buffer. IKBα (cat#4814) and P-IKBα (cat#9246) antibodies were obtained from Cell Signaling and used at a dilution of 1:1000.

### Western blot

For histone H2B and ubiquitinated H2B analyses, cell lines and patient samples were treated with 50 nM and 5 nM of Bortezomib, respectively, unless otherwise indicated in the manuscript. These concentrations were chosen based on the IC50 of bortezomib which differs significantly between cell lines and patient samples. Histones were extracted as previously described and blotted with anti-H2B (Millipore 07-371; 1:3000 dilution), anti-H2Bub11 (Millipore 05-1312 clone 56; 1:4000 dilution), anti-acetyl Histone H3 (Millipore 07-360; 1:1000 dilution), and anti-H3 (Millipore 04-928, 1:1000 dilution)^44^.

### Gene expression profiling and RNA sequencing

100 ng of total RNA was processed according to the Affymetrix GeneChip Whole Transcript Labeling protocol. 5.5 µg of biotinylated cDNA was fragmented and hybridized to Affymetrix GeneChip® Human Gene 2.0 ST arrays, then stained and scanned according to the manufacturer’s instructions. Main-design transcripts were used in the analysis with the cross-hybridized transcripts removed. The transcript signals were RMA-normalized (Robust Multi-array Average) and for gene set enrichment analysis (GSEA), the transcript signals were collapsed to signals of the genes. Differentially expressed genes were identified using the Limma R package^45^. RNA sequencing and analysis of patient samples was performed as previously described^46^. Transcript expression levels for gene expression analyses were estimated from RNA sequencing data as Fragments per Kilobase of transcript per Million mapped fragments (FPKM) as previously described^46^.

### Chromatin immunoprecipitation and sequencing (ChIP-Rx)

SEM cells were treated with 50 nM bortezomib or media for 0, 2, 4, and 6 h at 37 °C. Cells were cross-linked as previously described with 1% (H3K79me2) or 0.4% (H2B-ub) formaldehyde and snap frozen^47^. ChIP with reference exogenous genome (ChIP-Rx) using *Drosophila* S2 cells was done as previously described to allow for genome-wide quantitative comparisons of histone modification status across cell populations using defined quantities of a reference epigenome^48^. Library preparation, and sequencing was performed as previously described and is described below (https://www.encodeproject.org/documents/eaafee87-23f3-4bb4-adb1-585af01db120 and http://myers.hudsonalpha.org/documents).

Chromatin was fragmented in 600 µl aliquots with 3 pulses of 10 s, using a Soniprep 150. Following sonication drosophila chromatin was spiked in at a ratio of 2 µg SEM chromatin to 2.5 µg drosophila chromatin. 1 × 10^6^ cell equivalents were removed for whole chromatin extract (WCE) controls. Antibodies specific for H3K79me2 (12.8 µg/ml lysate, Abcam #ab3594) or H2B-ub (10 µg/ml lysate, Millipore #17-650) were used for immunoprecipitations. Samples were diluted to 1 × 10^7^ cell equivalents/ml and incubated with antibodies overnight at 4 °C. ChIP dilution buffer used during H2B-ub ChIP had an increase in NaCl concentration to 600 mM. Immune complexes were recovered by addition of 20 µl protein A bead slurry (Invitrogen #101141) and 10 µl protein G bead slurry (Pierce #20398), per 1 ml of lysate, for 1.5 h at 4 °C with agitation. Immunoprecipitation was verified by qPCR, using primers provided with antibodies and 10 ng of DNA. Fold difference from WCE was determined. ChIP DNA (10 ng of 200–400 bp fragments) was prepared for sequencing as described by Myers (http://myers.hudsonalpha.org/documents) with the following modification. After adapter ligation, DNA fragments ranging in size from 250–400 bp were gel purified using a minELUTE gel extraction kit (Qiagen). The resulting DNA library was sequenced on the Illumina Genome Analyzer.

Single-end reads of 50 bp were mapped to the human genome hg19 (GRCh37-lite) and fruit fly genome dm6 (D. melanogaster, UCSC BDGP Release 6) hybrid by BWA (version 0.7.12-r1039, default parameter)^49^. Duplicated reads were then marked with biobambam2 (version v2.0.87)^50^. After marking duplicated reads, bam files were then split by either human or fruit fly spike-in. For spike-in bam files, we counted the non-duplicated reads by samtools. For human bam files, only non-duplicated reads were kept by samtools (parameter “-q 1 -F 1024” version 1.2) for subsequent analysis^51^. We followed ENCODE guidelines for quality control of our data, details, and codes can be found in our previously publications^52,53^. After the quality was confirmed, we extended reads to fragment size ((detected by SPP v1.1) and generated bigwig tracks for visualization^54^. The bigwig tracks were normalized to 1 M uniquely mapped spike-in reads, e.g., we doubled the track height for human reads if the sample had 500k uniquely mapped spike-in reads. All samples had >30 M human reads and >10% of spike-in reads so we have confidence that the low count variation was well controlled. Correlation plots by multiBigwigSummary from deeptools (v3.0.2-1-ac19361) confirmed the libraries to be more similar between samples for the same antibody and different from the INPUTs (Supplementary Fig. 18)^55^. Heatmaps were generated by deeptools for putative fusion target genes in the SEM cell line as identified by Guenther and colleagues^56^. We then counted extended reads at the gene body or transcriptional start site (TSS) to 20% into the gene body. After TMM normalization we perform Empirical Bayes Statistics test after linear fitting from voom packge (R 3.23, edgeR 3.12.1, limma 3.26.9) to find differential binding sites^57^. Results are shown in Supplementary Data 4. We observed a high correlation of log2 fold change in H2Bub1 and H3K79me2 following exposure to bortezomib (Pearson Correlation 0.855, R2 = 0.495).

### CUT&RUN qPCR

SEM cells were treated with 50 nM bortezomib or media for 0, 2, 4, and 6 h at 37 °C. CUTANA^TM^ CUT&RUN Kit (EpiCypher 14-1048) was used, according to manufacturer’s instructions, to immunoprecipitate chromatin bound by MLL (Bethyl A300-086A), H2Bub1 (Cell Signaling 5546T), and normal rabbit IgG (CUTANA^TM^ CUT&RUN kit negative control). qPCR for MEIS1 exon 1 and CDK6 exon 1 was performed using PowerUP^TM^ SYBR^TM^ Green Master Mix (ThermoFisher A25742). Fold enrichment was calculated relative to the normal rabbit IgG control and then normalized to 0 h. MEIS1 exon1 primers: Forward GGAGCGCTTTTATGCTCAGT Reverse ATCCCTTAACGTCTCCAGCA. CDK6 exon1 primers: TTATCCTCCTCCCGTCTCCTCCT Reverse CTCGAAGCGAAGTCCTCAAC.

### Proteomic profiling by tandem mass tag labeling and mass spectrometry

Proteomic analysis was performed with a protocol of multiplexed tandem mass tag labeling, two-dimensional liquid chromatography and tandem mass spectrometry (TMT-LC/LC-MS/MS)14. TMT 10-plex isobaric label reagent set (Thermo Scientific) was used for proteome quantification of 10 samples simultaneously. 1 mg of protein per sample was used as starting material. Cells were lysed using a buffer that contains 50 mM HEPES, pH 8.5, 8 M urea, and 0.5% sodium deoxycholate. The lysate was split into three aliquots: one to measure the protein concentration, one for a positive control validation of H2B ubiquitination by western blotting, and one for proteomic analysis. Protein concentration of sample lysates were quantified by BCA protein assay (Thermo Fisher Scientific) with titrated BSA as a standard. ∼1 mg proteins per sample were first digested with Lys-C (Wako, 1:100 w/w) at room temperature for 2 hr, diluted 4 times with 50 mM HEPES, pH 8.5, and then further digested with trypsin (Promega, 1:100 w/w) overnight at room temperature. 1% trifluoroacetic acid was added to quench the digestion reaction, followed by desalting with Sep-Pak C18 cartridge (Waters), and the desalted peptides were dried by speedvac. Samples were then resuspended in 50 mM HEPES, pH 8.5, and were labeled with 10-plex TMT reagents following the manufacturer’s instruction. Lastly, 10 isobaric labeled samples were pooled together with equal amount, desalted again by Sep-Pak C18 cartridge and then speedvac dried. TMT-labeled and unlabeled samples were verified by LC-MS/MS. The basic pH reverse phase liquid chromatography peptides pre-fractionation were performed on Agilent 1220 LC system as previously described^58^. The analysis was carried out based on our optimized platform as previously described (Bai et al., 2017; Tan et al., 2017). The dried peptide fractions were reconstituted in loading buffer (5% formic acid), loaded on a reverse phase column (75 μm ×  50 cm, 1.9 μm C18 resin (Dr. Maisch GmbH, Germany)) and interfaced with a FUSION or Q Exactive HF mass spectrometer (Thermo Fisher Scientific). Peptides were eluted up to 6 hr by a 15-65% gradient of buffer B. (buffer A: 0.2% formic acid, 5% DMSO; buffer B: buffer A plus 65% acetonitrile, flow rate: 0.25 μl/min). A butterfly portfolio heater (Phoenix S&T) was applied to heat the column at 65 °C to reduce backpressure. The mass spectrometer was operated in data-dependent mode with MS1 settings of 60,000 resolution, 1 × 106 AGC target and 50 ms maximal ion time and top 20 MS/MS high-resolution scans with MS2 settings of 1 *m*/*z* isolation window with offset 0.2, 60,000 resolution, 100 ms maximal ion time, 1 × 105 AGC target, HCD, 33 normalized collision energy, and 40 s dynamic exclusion (35 normalized collision energy and 20 s dynamic exclusion for phosphoproteome). Peptide identification was performed using the JUMP search engine^59^. Commercially available database search engines can be divided into two categories: tag-based De novo sequencing (e.g., PEAKS with limited sensitivity) and pattern-based database search (e.g., SEQUEST, MASCOT). The JUMP software integrates these two methods to score putative peptides, showing significant improvements compared with these commercially available tools^59^. The JUMP software has previously been utilized in numerous publications^60^. Analysis was done similarly as previously described^61^, MS/MS raw files were first converted into mzXML format and searched against a composite target/decoy database FDR estimation. The target protein database was compiled from the Uniprot mouse and human database (Human database: 88,965 protein entries; Mouse database: 52,738 protein entries, downloaded in February 2015), the decoy database was generated by reversing target protein sequences. Spectra were searched with ±10 ppm mass tolerance for both precursor ions and product ions with fully tryptic restriction, static modification for TMT tag on N-terminus and lysine (+229.16293), dynamic modification for serine, threonine, and tyrosine (+79.96633, for phosphoproteome analysis), three maximal modification sites, two maximal missed cleavages, and the assignments of a, b, and y ions. Peptide spectrum matches (PSM) were first filtered by MS mass accuracy (∼2 ppm, ±4 standard deviations). PSMs of doubly charged peptides with JUMP Jscore of >30 were applied for global mass recalibration prior to the filtering. The survived PSMs were first grouped by precursor ion charge state and then further filtered by Jscore and dJn values. Cutoffs were applied on these values and were adjusted until a protein FDR < 1% was achieved. If one peptide was shared by multiple proteins, the protein with the highest PSM will represent the peptide according to the rule of parsimony^62^. For quantitation, an analysis was carried out in the following steps similarly as previously reported^61^. (i) TMT reporter ion intensities of each PSM were extracted; (ii) the raw intensities were corrected according to isotopic distribution of each labeling reagent^61^; (iii) PSMs with very low reporter ion intensities were excluded (e.g., minimum intensity <1000 and median intensity <5000); (iv) sample loading bias was corrected by normalization with the trimmed median intensity of all PSMs; (v) the mean-centered intensities across samples were calculated^61^; (vi) protein or phosphosite relative intensities were summarized by averaging related PSMs; (vii) protein or phosphosite absolute intensities were derived by multiplying the relative intensities by the grand-mean intensity of the top three most highly abundant PSMs.

### Aggressome immunofluorescence

SEM cells were cultured overnight before treatment. Bortezomib (Selleckchem) and Vorinostat (Selleckchem) were added to the culture medium for a final concentration of 3 nM Bortezomib, 1 μM Vorinostat, or both. 3 nM of bortezomib was used in combination with vorinostat in order to have viable cells to evaluate for aggressome immunofluorescence and minimize autofluorescence. After an 8-h incubation at 37 °C, the cells were fixed for 20 min in 4% paraformaldehyde (Electron Microscopy Sciences) at room temperature, permeabilized 10 min in 0.2% Triton™ X-100 and blocked in blocking buffer (1x PBS, 1% goat serum, 0.03% Triton™ X-100) for 1 h at room temperature. The cells were incubated in 2.0 μg/mL Anti-acetyl-alpha tubulin (Millipore Sigma, clone 6-11b-1), 1:500 Vimentin Rabbit Monoclonal Antibody (ThermoFisher Scientific clone MA5-16409), or both overnight at 4 °C for antibody labeling. Goat anti-Mouse IgG Secondary Antibody, Alexa Fluor 488 (ThermoFisher cat#A-11001), for alpha tubulin, and Goat anti-Rabbit IgG Secondary Antibody, Alexa Fluor 555 (ThermoFisher Scientific cat#A32732), for vimentin, were used at 2 μg/mL in 1X PBS, 1% serum, 0.03% Triton X-100 for 2 h at room temperature. The nuclei were stained with DAPI (ThermoFisher Scientific) for 5 min. The cells were washed and mounted on slides using Prolong Diamond (ThermoFisher Scientific). After 72 h, the slides were sealed and examined.

### Treatment of relapsed/refractory patients

Pediatric patients with relapsed/refractory KMT2Ar leukemia who were not eligible for a clinical trial received salvage chemotherapy regimens containing bortezomib, an anthracycline and vorinostat as previously published for hematologic malignancies on a compassionate basis as detailed in Supplementary Table 5^33,63–66^. Cases were reviewed following IRB approval at St. Jude Children’s Research Hospital. Written informed consent for chemotherapy was obtained from the parents or guardians at the time of treatment according to CARE guidelines and in compliance with the Declaration of Helsinki principles. Participants did not receive compensation.

### Statistics and reproducibility

#### Sample size

Inclusion criteria for preclinical studies: (1) patients less than one year of age at diagnosis (2) a diagnosis of acute lymphoblastic leukemia (3) the presence of a KMT2A rearrangement and (4) greater than 2 vials of banked specimens. 13 unique patients were identified that met the inclusion criteria, two of which also had banked relapsed specimens (see Supplementary Table 1). All 15 specimens were assayed for growth in vitro following NSG in vivo expansion and 6 specimens demonstrated > 1-fold increase in growth at 72 h (see Supplementary Fig. 1D). These 6 specimens were used for the high throughput drug screen and secondary dose–response curve validation. All 15 specimens were assayed for in vitro sensitivity to a subset of drugs. Downstream studies utilized a combination of patient specimens and the KMT2A rearranged infant ALL cell line SEM. Samples were chosen with a preference for cells that demonstrated growth at 72 h and had sufficient banked specimens for the assay.

Inclusion criteria for clinical treatment: Patients receiving care at St. Jude Children’s Research Hospital who had a diagnosis of KMT2A rearranged acute leukemia who were multiply relapsed or refractory to treatment were offered salvage regimens on a compassionate use basis.

#### Statistical analysis and reproducibility

High throughput drug screening aggregation of compound activities (median, coefficient of variation) across specimens was performed in R v3.6.1. Differential binding sites (ChIP-Rx), differential expression of proteins, and differential expression of RNA were determined by empirical Bayes statistics after linear fitting using limma v3.26.9. Combinatorial drugging experiments were analyzed using Bivariate Response to Additive Interacting Doses (BRAID) as previously described^30^. Murine Kaplan–Meier survival curves were plotted in Graph Pad Prism v9 and analyzed by Mantel–Cox log-rank test. Comparisons were performed by one-way ANOVA with multiple comparisons (proteomics dataset) and t test. No data were excluded from the analyses. The experiments were not randomized and the investigators were not blinded to allocation during experiments and outcome assessment.

### Reporting summary

Further information on research design is available in the Nature Portfolio Reporting Summary linked to this article.

## Supplementary information

Supplementary Information

Description of Additional Supplementary Files

Supplementary Data 1-20

Reporting Summary

## Data Availability

ChIP sequencing and gene expression data are publicly available in the GEO repository, accession GSE152816. Results from proteome profiling by TMT are included in the supplementary materials and have also been deposited to the ProteomeXchange Consortium via the Proteomics IDEntifications Database (PRIDE) partner repository that is publicly available with the dataset identifier PXD038845: https://www.ebi.ac.uk/pride^67,68^. Source data are provided as a Source data file. The remaining data are available within the article, supplementary information, or source data file. Correspondence and material requests should be addressed to Tanja A. Gruber: tagruber@stanford.edu. Source data are provided with this paper.

## Source data

Source Data
