# Author Correction: Proteasome inhibition targets the KMT2A transcriptional complex in acute lymphoblastic leukemia

**DOI:** 10.1038/s41467-023-37141-4

**Published:** 2023-03-09

**Authors:** Jennifer L. Kamens, Stephanie Nance, Cary Koss, Beisi Xu, Anitria Cotton, Jeannie W. Lam, Elizabeth A. R. Garfinkle, Pratima Nallagatla, Amelia M. R. Smith, Sharnise Mitchell, Jing Ma, Duane Currier, William C. Wright, Kanisha Kavdia, Vishwajeeth R. Pagala, Wonil Kim, LaShanale M. Wallace, Ji-Hoon Cho, Yiping Fan, Aman Seth, Nathaniel Twarog, John K. Choi, Esther A. Obeng, Mark E. Hatley, Monika L. Metzger, Hiroto Inaba, Sima Jeha, Jeffrey E. Rubnitz, Junmin Peng, Taosheng Chen, Anang A. Shelat, R. Kiplin Guy, Tanja A. Gruber

**Affiliations:** 1 Department of Pediatrics, Stanford University School of Medicine, Stanford, CA USA; 2 Department of Oncology, St. Jude Children’s Research Hospital, Memphis, TN USA; 3 Department of Computational Biology, St. Jude Children’s Research Hospital, Memphis, TN USA; 4 The University of Tennessee Health Science Center, Memphis, TN USA; 5 Department of Pathology, St. Jude Children’s Research Hospital, Memphis, TN USA; 6 Department of Chemical Biology and Therapeutics, St. Jude Children’s Research Hospital, Memphis, TN USA; 7 Center for Proteomics and Metabolomics, St. Jude Children’s Research Hospital, Memphis, TN USA; 8 Department of Pathology, University of Alabama School of Medicine, Birmingham, AL USA; 9 Department of Structural Biology, St. Jude Children’s Research Hospital, Memphis, TN USA; 10 Department of Developmental Neurobiology, St. Jude Children’s Research Hospital, Memphis, TN USA; 11 Department of Pharmaceutical Sciences, University of Kentucky, Lexington, KY USA; 12 Stanford Cancer Institute, Stanford University School of Medicine, Stanford, CA USA

Correction to: *Nature Communications* 10.1038/s41467-023-36370-x, published online 13 February 2023

In this article the author name William C. Wright was incorrectly written as Charlie Wright. The original article has been corrected.

