# Supplementary material for: Proteasome inhibition targets the KMT2A transcriptional complex in acute lymphoblastic leukemia
Source: Nat Commun. 2023 Feb 13;14:809. doi: 10.1038/s41467-023-36370-x (PMC9925443; doi:10.1038/s41467-023-36370-x)
Supplement: Supplementary file 1 — Supplementary Information [file 41467_2023_36370_MOESM1_ESM.pdf]

SUPPLEMENTARY INFORMATION FOR

Proteasome Inhibition Targets the KMT2A Transcriptional Complex in Acute Lymphoblastic Leukemia

Jennifer L. Kamens[1], Stephanie Nance[2], Cary Koss[2], Beisi Xu[3], Anitria Cotton[2], Jeannie W. Lam[1], Elizabeth A.R. Garfinkle[4], Pratima Nallagatla[1], Amelia M.R. Smith[1], Sharnise Mitchell[2], Jing Ma[5], Duane Currier[6], Charlie Wright[6], Kanisha Kavdia[7], Vishwajeeth R. Pagala[7], Wonil Kim[2], LaShanale M. Wallace[2], Ji-Hoon Cho[7], Yiping Fan[3], Aman Seth[5], Nathaniel Twarog[6], John K. Choi[8], Esther A. Obeng[2], Mark E. Hatley[2], Monika L. Metzger[2], Hiroto Inaba[2], Sima Jeha[2], Jeffrey E. Rubnitz[2], Junmin Peng[7,9-10], Taosheng Chen[6], Anang A. Shelat[6], R. Kiplin Guy[11], and Tanja A. Gruber[1,12*].

[1]Department of Pediatrics Stanford University School of Medicine, Stanford, CA, USA

[2]Department of Oncology St. Jude Children's Research Hospital, Memphis, TN, USA

[3]Department of Computational Biology St. Jude Children's Research Hospital, Memphis, TN, USA

[4]The University of Tennessee Health Science Center, Memphis, TN, USA

[5]Department of Pathology St. Jude Children's Research Hospital, Memphis, TN, USA

[6]Department of Chemical Biology and Therapeutics St. Jude Children's Research Hospital, Memphis, TN, USA

[7]Center for Proteomics and Metabolomics, St. Jude Children's Research Hospital, Memphis, TN, USA

[8]Department of Pathology University of Alabama School of Medicine, Birmingham, AL, USA

[9]Department of Structural Biology, St. Jude Children's Research Hospital, Memphis, TN, USA

[10]Department of Developmental Neurobiology, St. Jude Children's Research Hospital, Memphis, TN, USA

[11]Department of Pharmaceutical Sciences University of Kentucky, Lexington, KY, USA

[12]Stanford Cancer Institute, Stanford University School of Medicine, Stanford, CA, USA

*Corresponding Author:

Tanja Andrea Gruber, M.D. Ph.D.
Department of Pediatrics
Stanford University School of Medicine
tagruber@stanford.edu

Contents

Supplementary Tables 4
Supplementary Table 1: Patient Samples 4
Supplementary Table 2: Average IC_{50} of Select Compounds Across All Samples 6
Supplementary Table 3: Activity of HDAC Inhibitors in Infant ALL 7
Supplementary Table 4: HAT and HDAC Protein Level Changes Following Bortezomib 8
Supplementary Table 5: Salvage Chemotherapy Regimens 9
Supplementary Table 6: Adverse Events 10
Supplementary Data 11
Supplementary Data 1: FDA Approved Compounds Used in Screening 11
Supplementary Data 2: Activity of FDA Approved Compounds at 10uM in Infant ALL 11
Supplementary Data 3: Secondary Validation and IC50 Determination of Top Hits 11
Supplementary Data 4: Peak Calls KMT2A Targets ChIP-Rx 11
Supplementary Data 5: Differentially Expressed Genes in Bortezomib Treated Patient Samples .. 11
Supplementary Data 6: TMT Proteome Profiling 12
Supplementary Data 7: ANOVA Analysis Proteome Time Course 12
Supplementary Data 8: Differential Enrichment Proteome Analysis of Bortezomib Treated KMT2Ar Leukemia Cells 12
Supplementary Data 9: Enriched Hallmark Pathways at 6 Hours Following Exposure to Proteasome Inhibition in KMT2Ar Leukemia 13
Supplementary Data 10: Enriched Hallmark Pathways at 12 Hours Following Exposure to Proteasome Inhibition in KMT2Ar Leukemia 13
Supplementary Data 11: Enriched Hallmark Pathways at 16 Hours Following Exposure to Proteasome Inhibition in KMT2Ar Leukemia 13
Supplementary Data 12: Enriched Hallmark Pathways at 20 Hours Following Exposure to Proteasome Inhibition in KMT2Ar Leukemia 13
Supplementary Data 13: Enriched KEGG Pathways at 6 Hours Following Exposure to Proteasome Inhibition in KMT2Ar Leukemia 14
Supplementary Data 14: Enriched KEGG Pathways at 12 Hours Following Exposure to Proteasome Inhibition in KMT2Ar Leukemia 14
Supplementary Data 15: Enriched KEGG Pathways at 16 Hours Following Exposure to Proteasome Inhibition in KMT2Ar Leukemia 14
Supplementary Data 16: Enriched KEGG Pathways at 20 Hours Following Exposure to Proteasome Inhibition in KMT2Ar Leukemia 14
Supplementary Data 17: Enriched Reactome Pathways at 6 Hours Following Exposure to Proteasome Inhibition in KMT2Ar Leukemia 15

Supplementary Data 18: Enriched Reactome Pathways at 12 Hours Following Exposure to Proteasome Inhibition in KMT2Ar Leukemia.......... 15
Supplementary Data 19: Enriched Reactome Pathways at 16 Hours Following Exposure to Proteasome Inhibition in KMT2Ar Leukemia.......... 15
Supplementary Data 20: Enriched Reactome Pathways at 20 Hours Following Exposure to Proteasome Inhibition in KMT2Ar Leukemia.......... 15
Supplementary Figures 16
Supplementary Figure 1. Platform to Identify Active Agents in Infantile ALL.......... 16
Supplementary Figure 2. Cell titer glo as a surrogate for apoptosis.......... 17
Supplementary Figure 3. Active Antineoplastic Agents. 18
Supplementary Figure 4. Activity of HDAC Inhibitors.......... 19
Supplementary Figure 5. Activity of Proteasome Inhibitors in Infant ALL.......... 20
Supplementary Figure 6. Activity of Bortezomib in KMT2Ar Patient Derived Xenograft. 21
Supplementary Figure 7. NFkB Activity in KMT2Ar Leukemia Cells. 22
Supplementary Figure 8. Bortezomib Exposure Depletes H2Bub1.......... 23
Supplementary Figure 9. H2Bub1 of KMT2Ar Leukemia Following Exposure to Chemotherapy ... 24
Supplementary Figure 10. Bortezomib Exposure Depletes H2Bub1 and H3K79me2 at KMT2A Target Genes 25
Supplementary Figure 11. Wild type KMT2A Levels Following Proteasome Inhibition.......... 26
Supplementary Figure 12. Bortezomib Exposure Depletes KMT2A at Target Genes 27
Supplementary Figure 13. Global Protein Changes of KMT2r Leukemia Following Proteasome Inhibition. 28
Supplementary Figure 14. Differentially Expressed Proteins of KMT2Ar Leukemia Following Proteasome Inhibition. 29
Supplementary Figure 15. KMT2Ar Complex Accumulation and Proteotoxic Stress in Bortezomib Treated Cells.......... 30
Supplementary Figure 16. Histone Acetylation in Bortezomib and Vorinostat Treated Cells.......... 31
Supplementary Figure 17. Combinatorial Treatment of KMT2Ar Leukemia with Bortezomib and Vorinostat.......... 32
Supplementary Figure 18. ChIP-Rx Correlation Plots.......... 33
Supplementary References.......... 34

Supplementary Tables

Supplementary Table 1: Patient Samples

Sample ID*	Immunophenotype	Age at Diagnosis	KMT2A Fusion	Non-Silent Mutations[†]
INF001D	Pre-B ALL	69 days	KMT2A-AFF1	KRAS^{G12D}
INF001R	Pre-B ALL	69 days	KMT2A-AFF1	chr4p16.3--q22.1^{AMP}<br>chr7q11.1--q36.3^{AMP}<br>chr11q23.3--q25^{AMP}<br>chr2p23.3^{DEL}<br>chr7p22.3--p11.2^{DEL}<br>DCAF10^{R456C}<br>KRAS^{G12D}<br>LSG1^{D317N}<br>NCKAP1^{D262N}<br>ODF1^{S163L}<br>RBMXL3^{L930I}
INF002D	Pre-B ALL	212 days	KMT2A-AFF1	chrXp22.33--q28^{AMP} TFPI^{N20H}
INF002R	Pre-B ALL	212 days	KMT2A-AFF1	RAP1A^{DEL}<br>APC2^{DEL}<br>chr2p11.2^{AMP}<br>chrXp22.33--q28^{AMP}<br>chr1p32.3^{AMP}<br>chr1p13.2^{DEL}<br>chr19p13.3^{DEL}<br>chr2q37.1^{DEL}<br>chr7q22.1^{DEL}<br>chr9p13.3^{DEL}<br>chr19p13.3^{DEL}<br>ARNTL2^{S539L}<br>AXL^{D396Y}<br>COPG^{V637M}<br>DNHD1^{R2645C}<br>EPHB1^{V391I}<br>HEATR3^{V166L}<br>KCNAB1^{D44E}<br>KCND3^{R139C}<br>KPNA7^{F53L}<br>NEB^{L3626S}<br>NOX3^{S332L}<br>PAX5^{G304V}<br>PCDHGA5^{D666N}<br>PTPRO^{R978H}<br>PUM1^{N489D}<br>SLCO3A1^{F368L}<br>SREBF2^{D414E}<br>THBS2^{T1074M}<br>TNN^{V369M}<br>TRAPPC9^{R651Q}<br>TTLL4^{R625Q}<br>VPRBP^{R1104Q}<br>ZNF443^{T220I}
INF003D	Pre-B ALL	237 days	KMT2A-AFF1	chr7q22.1^{AMP}<br>chr15q25.2^{AMP}<br>chr19p13.3^{AMP}<br>GHDC^{T354R}

Sample ID*	Immunophenotype	Age at Diagnosis	KMT2A Fusion	Non-Silent Mutations†
INF005D	T ALL	362 days	KMT2A-MLLT1	chr9p21.3^{DEL}<br>chr13q14.2;q14.3^{DEL}<br>chr4q22.1^{DEL}<br>DENND5B^{R1023K}<br>PCDH19^{A535S}
INF006D	Early Pre-B ALL	60 days	KMT2A-AFF1	chrXp22.33--q28^{AMP}
INF013D	Early Pre-B ALL	336 days	KMT2A-AFF1	chr19p13.3^{DEL}<br>chr17p11.2^{DEL}<br>chr15q24.1^{DEL}<br>chr17p13.3^{DEL}<br>chr17p11.2^{DEL}<br>chr8q22.2^{DEL}<br>chr17p11.2^{DEL}<br>JAG1^{Q1118K}<br>SIPA1L3^{A732_F735del}<br>TP53^{A276_R280del}
INF014D	Early Pre-B ALL	104 days	KMT2A-AFF1	chr4q28.1^{DEL}<br>TMEM82^{T150S}
INF016D	Pre-B ALL	31 days	KMT2A-MLLT1	chrXp22.33--q28^{AMP}<br>XBP1^{A171_P172fs}
INF017D	Pre-B ALL	166 days	KMT2A-AFF1	chr2q37.1^{DEL}<br>MAMDC2^{T240M}<br>PIK3C2B^{L487F}
INF019D	Early Pre-B ALL	288 days	KMT2A-MLLT3	chr9p13.2^{DEL}<br>chr15q25.1^{DEL}<br>chr1p31.1;p22.3^{DEL}<br>KRAS^{G12V}<br>FABP12^{S138*}<br>DCAF5^{L296fs}
INF021D	Pre-B ALL	304 days	KMT2A-MLLT3	chr9p21.3--p13.2^{DEL}<br>chr11q23.3^{DEL}<br>chr11q23.3;q24.1^{DEL}<br>AQP12B^{R55T}<br>NRAS^{G13V}<br>PLXNA2^{R141W}<br>AHDC1^{R789*}
INFRF01R	Early Pre-B ALL	105 days	KMT2A-AFF1	COL6A1^{E197V}<br>TSGA10IP^{Q272R}<br>HAUS3^{K140_E141del}<br>PIK3CD$^{D336>VD}$
INFRF02D	Early Pre-B ALL	64 days	KMT2A-MLLT1	None

*D designates sample obtained at diagnosis, R designates sample obtained at relapse

†All samples underwent whole genome sequencing. Non-silent SNVs in coding regions of genes and copy number alterations are shown.

Supplementary Table 2: Average IC_{50} of Select Compounds Across All Samples

Drug Name	IC_{50} µM*	Samples with No IC_{50}†	Childhood ALL§
Artesunate	10.54 (3.5-17)	0	
Bendamustine	19.31 (4.2-52)	0	
BEZ-235	1.29 (0.096-4.2)	1	
Bortezomib	0.0016 ($1.6x10^{-11}$-0.0058)	0	0.046-1.34 µM
Carfilzomib	0.0009 (0.0002-0.0021)	4	
Clofarabine	0.022 (0.0027-0.099)	0	0.015-0.046 µM
Cytarabine	3.59 (0.12-13)	0	0.576-3.74 µM
Daunorubicin	0.032 (0.00077-0.12)	0	0.0053-1.05 µM
Decitabine	32.14 ($1.4x10^{-11}$-200)	8	
Dihydroartemisinin	10.106 (1.5-15)	1	
EPZ-5676	NA	15	
Doxorubicin	0.132 (0.0012-0.8)	0	0.054-4.35 µM
Etoposide	8.189 (0.42-16)	0	1.5-10.16 µM
Ganetespib	0.027 (0.0013-0.085)	1	
Idealisib	12.41 (0.029-24)	4	
JQ1	5.22 (0.05-7.9)	0	
Mercaptopurine	11.73 (0.25-27)	4	
Methotrexate	0.011 (NA, 1 specimen)	14	
Methylprednisolone	4.68 (0.0014-28)	3	
Mitoxantrone	0.128 (0.00001-1.1)	0	0.001-0.92 µM
MLN 4924	6.39 (0.015-20)	1	
Oprozomib	0.042 (0.0073-0.1)	1	
Panobinostat	0.224 ($6x10^{-17}$-1.1)	0	
PD-0325901	3.36 (0.0013-25)	1	
Prednisolone	10.6 (1.7-15)	5	3.88-332.9 µM
Quizartinib	2.49 (0.033-9.5)	5	
Sorafenib	14.83 (1-27)	9	
Thioguianine	3.87 (0.62-15)	0	17.9-57.42 µM
Torin1	1.54 (0.005-8.2)	2	
Vincristine	0.66 (0.0019-4.9)	1	0.085-0.76 µM
Vionorelbine	4.97 (0.051-15)	1	
Vorinostat	1.08 (0.013-9.1)	0	0.55-1.78 µM

*Average IC_{50} in µM and range for patient samples (N=15 patient specimens assayed) that had a response to the compound.

†Number of samples that did not respond to the compound precluding IC_{50} determination.

§IC_{50} ranges for primary childhood ALL samples as previously published[1, 2]. Range for vorinostat as reported by the pediatric preclinical testing program which included four pediatric ALL cell lines and one primary patient specimen[3].

Supplementary Table 3: Activity of HDAC Inhibitors in Infant ALL

Compound	Group	Specificity	Median IC_{50} nM	FDA Approved	Pediatric Data*
QUISINOSTAT	HDAC Class I/II Inhibitors	HDAC Class I/II Pan Inhibitor	8.3	No	No
**PANOBINOSTAT**		**HDAC Class I/II Pan Inhibitor**	**13.81**	**Yes**	In progress
DACINOSTAT		HDAC Class I/II Pan Inhibitor	28.99	No	No
GIVINOSTAT		HDAC Class I/II Pan Inhibitor	269.9	No	In progress
S-HDAC-42		HDAC Class I/II Pan Inhibitor	309.7	No	No
**BELINOSTAT**		**HDAC Class I/II Pan Inhibitor**	**347.1**	**Yes**	**No**
ABEXINOSTAT		HDAC Class I/II Pan Inhibitor	368.2	No	No
**PRACINOSTAT**		**HDAC Class I/II Pan Inhibitor**	**450.7**	**Yes**	**No**
TRICHOSTATIN A		HDAC Class I/II Pan Inhibitor	499.5	No	No
M344		HDAC Class I/II Pan Inhibitor	974.5	No	No
SCRIPTAID		HDAC Class I/II Pan Inhibitor	1058	No	No
**VORINOSTAT**		**HDAC Class I/II Pan Inhibitor**	**1260**	**Yes**	**Yes (Phase 1/2)**
RESMINOSTAT		HDAC 1/3/6 Inhibitor	1419	No	No
BRD73954		HDAC 6/8 Inhibitor	37360	No	No
**ROMIDEPSIN**	HDAC Class I Inhibitors	**HDAC Class I Pan Inhibitor**	**3.29**	**Yes**	**Yes (Phase 1)**
OKI-5		HDAC Class I Pan Inhibitor	30.72	No	No
CHIDAMIDE		HDAC Class I Pan Inhibitor	729.9	No	No
MOCETINOSTAT		HDAC Class I Pan Inhibitor	996.8	No	No
ENTINOSTAT		HDAC Class I Pan Inhibitor	1199	No	No
PCI 34051		HDAC 8 Inhibitor	2457	No	No
4SC-202		HDAC Class I Pan Inhibitor	2845	No	No
MI 192		HDAC Class I Pan Inhibitor	3522	No	No
RG2833 (RGFP109)		HDAC Class I Pan Inhibitor	4201	No	No
TACEDINALINE		HDAC Class I Pan Inhibitor	5258	No	No
RGFP966		HDAC 3 Inhibitor	5583	No	No
BML-281	HDAC Class II Inhibitors	HDAC 6 Inhibitor	622.5	No	No
LMK-235		HDAC 4/5 Inhibitor	731.5	No	No
ROCILINOSTAT		HDAC 6 Inhibitor	1845	No	No
TASQUINIMOD		HDAC 4 Inhibitor	33000	No	No
TMP269		HDAC Class II Pan Inhibitor	33300	No	No

*Compounds that have been evaluated in pediatric clinical trials

Supplementary Table 4: HAT and HDAC Protein Level Changes Following Bortezomib

Protein	Class	Subclass	Substrate Specificity*	Protein Level†	p value§	FDR§
EP300	HAT	p300/CBP	H2A,H2B,H3,H4	NSC	0.50	0.53
CREBBP	HAT	p300/CBP	H2A,H2B,H3,H4	NSC	0.27	0.31
KAT8	HAT	MYST	H4 (H2A,H3)	Decrease	0.003	0.01
KAT7	HAT	MYST	H3,H4	NSC	0.20	0.24
KAT6A	HAT	MYST	H3,H4	Increase	0.04	0.07
KAT6B	HAT	MYST	H3,H4	Decrease	0.002	0.01
KAT5	HAT	MYST	H2A,H4 (H3)	Increase	0.058	0.09
KAT2A	HAT	GNAT	H3, (H4,H2B)	Decrease	0.05	0.08
KAT2B	HAT	GNAT	H3,H4	NSC	0.06	0.09
HAT1	HAT	GNAT	H4 (H2A)	Decrease	0.02	0.04
HDAC1	HDAC	Class I	Func Redundant	Decrease	0.02	0.04
HDAC2	HDAC	Class I	Func Redundant	Decrease	0.02	0.04
HDAC3	HDAC	Class I	Func Redundant	NSC	0.24	0.28
HDAC8	HDAC	Class I	Func Redundant	NSC	0.61	0.63
HDAC4	HDAC	Class IIa	Func Redundant	NSC	0.43	0.46
HDAC5	HDAC	Class IIa	Func Redundant	NSC	0.22	0.26
HDAC7	HDAC	Class IIa	Func Redundant	NSC	0.35	0.38
HDAC7 Isoform 10	HDAC	Class IIa	Func Redundant	NSC	0.08	0.11
HDAC9 Isoform 11	HDAC	Class IIa	Func Redundant	NSC	0.09	0.12
HDAC9 Isoform 16	HDAC	Class IIa	Func Redundant	NSC	0.10	0.13
HDAC9	HDAC	Class IIa	Func Redundant	NSC	0.11	0.15
HDAC6	HDAC	Class IIb	Func Redundant	NSC	0.07	0.10
HDAC10	HDAC	Class IIb	Func Redundant	Decrease	0.05	0.08
SIRT1	HDAC	Class III	H3,H4,H1	Decrease	0.02	0.04
SIRT2	HDAC	Class III	H3,H4	NSC	0.10	0.14
SIRT3	HDAC	Class III	H4	NSC	0.14	0.17
SIRT5	HDAC	Class III	None	NSC	0.75	0.76
SIRT6	HDAC	Class III	H3	Decrease	0.05	0.07
SIRT7	HDAC	Class III	H3	Decrease	0.005	0.02

*Func Redundant – functional redundancy.
†Change in protein level following exposure to bortezomib over 20 hours. See supplementary table 8.
NSC – no significant change
§As determined by ANOVA.

Supplementary Table 5: Salvage Chemotherapy Regimens

			Treatment (days chemo administered)*†					
Case‡π	Age at Dx	Age at Tx§	Bortezomib	Vorinostat	Mitoxantrone	Dexamethasone	PEG Asparaginase	Vincristine
1	106 days	7 months	1,4,8,11	NA	1,2	1-5, 15-19	3,18	3,10,17,24
2	65 days	5 months	1,4,8,11	NA	1,2	1-5, 15-19	3,18	3,10,17,24
3a	54 days	8 months	1,4,8,11	NA	1,2	1-5, 15-19	3,18	3,10,17,24
3b	54 days	18 months	1,4,8,11,15,18, 22,25	1-4, 8-11, 15-18	1,2	1,2,4,5,8,9,11,12, 15,16,18,19	3,18	NA
4	9.9 years	11 years	1,4,8,11,15,18	1-4, 8-11, 15-18	1,2	1,2,4,5,8,9,11,12, 15,16,18,19	3,18	NA
5	2.3 years	3.5 years	1,4,8,11,15,18, 22,25	1-4, 8-11, 15-18	1,2	NA	NA	NA
6	10 months	1.5 years	1,4,8,11,15,18, 22,25	1-4, 8-11, 15-18	1,2	NA	NA	NA
7	7.3 years	9 years	1,4,8,11,15,18	1-4, 8-11, 15-18	1,2	NA	NA	NA
8	1.3 years	2.3 years	1,4,8,11,15,18, 22,25	1-4, 8-11, 15-18, 22-25	1,2	NA	NA	NA
9	2 months	4 years	1,4,8,11,15,18,	1-4, 8-11, 15-18	1,2	1-4, 8-11, 15-18, 22-25	3,18	NA
10	7.5 months	14 months	1,4,8,11,15,18, 22,25	1-4, 8-11, 15-18, 22-25	1,2	1-4, 8-11, 15-18, 22-25	3,18	NA

*All patients received intrathecal methotrexate, hydrocortisone, cytarabine (“MHA”); days of administration for each agent varied between patients.

†Dosing: Bortezomib 0.043mg/kg/dose patients <1 year of age and/or <10kg, 1.3mg/m2/dose for patients ≥1 year of age AND ≥10kg; Vorinostat 180mg/m2/dose; Mitoxantrone 0.33mg/kg/dose patients <1 year of age and/or <10kg, 10mg/m2/dose for patients ≥1 year of age AND ≥10kg; Dexamethasone 20mg/m2/day divided BID case 1-3a, 10mg/m2/day divided BID case 3b,4,9-10; PEG asparaginase 2500 units/dose; Vincristine 0.05mg/kg/dose.

‡*KMT2A-AF4* cases 1,4,9; *KMT2A-ENL* cases 2,3,10; *KMT2A-AF10* cases 8,6; *KMT2A-AF9* case 5; *KMT2A-AF6* case 7.

πMale gender cases 1,4,10; Female gender cases 2,3,5,6,7,8,9

§Age at the time the chemotherapy was administered

Supplementary Table 6: Adverse Events

Category	Description	Organism	Grade 2	Grade 3	Grade 4	Grade 5
Infections and infestations	Colitis	CDiff		2		
Infections and infestations	Mucosal infection	Candida		1		
Infections and infestations	Febrile Neutropenia	NA		2		
Infections and infestations	Sepsis	Pseudomonas			1	
Infections and infestations	Lung infection	Human metapneumovirus	1			
Infections and infestations	Upper respiratory infection	Rhinovirus	1			
Infections and infestations	Cellulitis	Unknown		1		
Infections and infestations	Urinary Tract Infection	Ecoli		1		
Infections and infestations	Viremia	Adenovirus			1	
Infections and infestations	Fungemia	Fusarium		1		
Laboratory Investigations	Alanine aminotransferase increased	NA		1		
Laboratory Investigations	Aspartate aminotransferase increased	NA		1		
Laboratory Investigations	Creatinine increased	NA	1			
Nervous system disorders	Recurrent laryngeal nerve palsy	NA	1			
Renal and urinary disorders	Urinary Retention	NA	1			
Vascular Disorders	Hypertension	NA		1		

Supplementary Data

Supplementary Data 1: FDA Approved Compounds Used in Screening

See excel table "Supplementary Data 1: FDA Approved Compounds Used in Screening"

Supplementary Data 2: Activity of FDA Approved Compounds at 10uM in Infant ALL

See excel table "Supplementary Data 2: Activity of FDA Approved Compounds at 10uM in Infant ALL"

Abbreviations:
CV – coefficient of variation
SE – standard error
ATC - Anatomical Therapeutic Chemical classification system identification number

Supplementary Data 3: Secondary Validation and IC50 Determination of Top Hits

See excel table "Supplementary Data 3: Secondary Validation and IC50 Determination of Top Hits"

qAC50 – standard potency estimates derived from concentration-response assays; 10^{-9} = 1nM
Sinf - extrapolated activity of the compound at an infinite concentration to determine if compound can reach 100% toxicity(-100) or if it is only capable of partial toxicity.

Supplementary Data 4: Peak Calls KMT2A Targets ChIP-Rx

See excel table "Supplementary Data 4: Peak Calls KMT2Ar Targets ChIP-Rx"

Abbreviations:
FPKM – fragments per kilobase million
log2fc – log2 fold change
Averlog2 – average log2
Regulation – Change in histone modification following exposure to bortezomib

KMT2Ar targets as defined by previously published ChIP-seq in the SEM cell line[4]

Supplementary Data 5: Differentially Expressed Genes in Bortezomib Treated Patient Samples

See excel table "Supplementary Data 5: Differentially Expressed Genes in Bortezomib Treated Patient Samples

Supplementary Data 6: TMT Proteome Profiling

See excel table "Supplementary Data 6: TMT Proteome Profiling"

Abbreviations:
GN – gene name
PSM# – the summed number of spectral counts (i.e. MS/MS scans) assigned to one identified protein
Total Peptide# – total number of peptides identified for a protein
Unique Peptide# - number of peptide sequences that are unique to a protein group
m/z – mass to charge ratio
z – charge
ppm – delta mass
Xcorr – cross correlation
dCN – delta correlation
q value – minimal false discovery rate
sig - the signal is based on TMT reporter ion intensity
FDR – false discovery rate

p-values and FDR were calculated for each protein across the entire time course by ANOVA. See also supplementary figure 12.

Supplementary Data 7: ANOVA Analysis Proteome Time Course

See excel table "Supplementary Data 7: ANOVA Analysis Proteome Time Course"

Abbreviations:
PSM# – the summed number of spectral counts (i.e. MS/MS scans) assigned to one identified protein
Total Peptide# – total number of peptides identified for a protein

p-values and FDR were calculated for each protein across the entire time course by ANOVA. See also supplementary figure 12.

Supplementary Data 8: Differential Enrichment Proteome Analysis of Bortezomib Treated KMT2Ar Leukemia Cells

See excel table "Supplementary Data 8: Differential Enrichment Proteome Analysis of Bortezomib Treated KMT2Ar Leukemia Cells"

Supplementary Data 9: Enriched Hallmark Pathways at 6 Hours Following Exposure to Proteasome Inhibition in KMT2Ar Leukemia

See excel table "Supplementary Data 9: Enriched Hallmark Pathways at 6 Hours Following Exposure to Proteasome Inhibition in KMT2Ar Leukemia"

Abbreviations:
ES – enrichment score
NES – normalized enrichment score

Supplementary Data 10: Enriched Hallmark Pathways at 12 Hours Following Exposure to Proteasome Inhibition in KMT2Ar Leukemia

See excel table "Supplementary Data 10: Enriched Hallmark Pathways at 12 Hours Following Exposure to Proteasome Inhibition in KMT2Ar Leukemia"

Abbreviations:
ES – enrichment score
NES – normalized enrichment score

Supplementary Data 11: Enriched Hallmark Pathways at 16 Hours Following Exposure to Proteasome Inhibition in KMT2Ar Leukemia

See excel table "Supplementary Data 11: Enriched Hallmark Pathways at 16 Hours Following Exposure to Proteasome Inhibition in KMT2Ar Leukemia"

Abbreviations:
ES – enrichment score
NES – normalized enrichment score

Supplementary Data 12: Enriched Hallmark Pathways at 20 Hours Following Exposure to Proteasome Inhibition in KMT2Ar Leukemia

See excel table "Supplementary Data 12: Enriched Hallmark Pathways at 20 Hours Following Exposure to Proteasome Inhibition in KMT2Ar Leukemia"

Abbreviations:
ES – enrichment score
NES – normalized enrichment score

Supplementary Data 13: Enriched KEGG Pathways at 6 Hours Following Exposure to Proteasome Inhibition in KMT2Ar Leukemia

See excel table "Supplementary Data 13: Enriched KEGG Pathways at 6 Hours Following Exposure to Proteasome Inhibition in KMT2Ar Leukemia"

Abbreviations:
ES – enrichment score
NES – normalized enrichment score

Supplementary Data 14: Enriched KEGG Pathways at 12 Hours Following Exposure to Proteasome Inhibition in KMT2Ar Leukemia

See excel table "Supplementary Data 14: Enriched KEGG Pathways at 12 Hours Following Exposure to Proteasome Inhibition in KMT2Ar Leukemia"

Abbreviations:
ES – enrichment score
NES – normalized enrichment score

Supplementary Data 15: Enriched KEGG Pathways at 16 Hours Following Exposure to Proteasome Inhibition in KMT2Ar Leukemia

See excel table "Supplementary Data 15: Enriched KEGG Pathways at 16 Hours Following Exposure to Proteasome Inhibition in KMT2Ar Leukemia"

Abbreviations:
ES – enrichment score
NES – normalized enrichment score

Supplementary Data 16: Enriched KEGG Pathways at 20 Hours Following Exposure to Proteasome Inhibition in KMT2Ar Leukemia

See excel table "Supplementary Data 16: Enriched KEGG Pathways at 20 Hours Following Exposure to Proteasome Inhibition in KMT2Ar Leukemia"

Abbreviations:
ES – enrichment score
NES – normalized enrichment score

Supplementary Data 17: Enriched Reactome Pathways at 6 Hours Following Exposure to Proteasome Inhibition in KMT2Ar Leukemia

See excel table "Supplementary Data 17: Enriched Reactome Pathways at 6 Hours Following Exposure to Proteasome Inhibition in KMT2Ar Leukemia"

Abbreviations:
ES – enrichment score
NES – normalized enrichment score

Supplementary Data 18: Enriched Reactome Pathways at 12 Hours Following Exposure to Proteasome Inhibition in KMT2Ar Leukemia

See excel table "Supplementary Data 18: Enriched Reactome Pathways at 12 Hours Following Exposure to Proteasome Inhibition in KMT2Ar Leukemia"

Abbreviations:
ES – enrichment score
NES – normalized enrichment score

Supplementary Data 19: Enriched Reactome Pathways at 16 Hours Following Exposure to Proteasome Inhibition in KMT2Ar Leukemia

See excel table "Supplementary Data 19: Enriched Reactome Pathways at 16 Hours Following Exposure to Proteasome Inhibition in KMT2Ar Leukemia"

Abbreviations:
ES – enrichment score
NES – normalized enrichment score

Supplementary Data 20: Enriched Reactome Pathways at 20 Hours Following Exposure to Proteasome Inhibition in KMT2Ar Leukemia

See excel table "Supplementary Data 20: Enriched Reactome Pathways at 20 Hours Following Exposure to Proteasome Inhibition in KMT2Ar Leukemia"

Abbreviations:
ES – enrichment score
NES – normalized enrichment score

Supplementary Figures

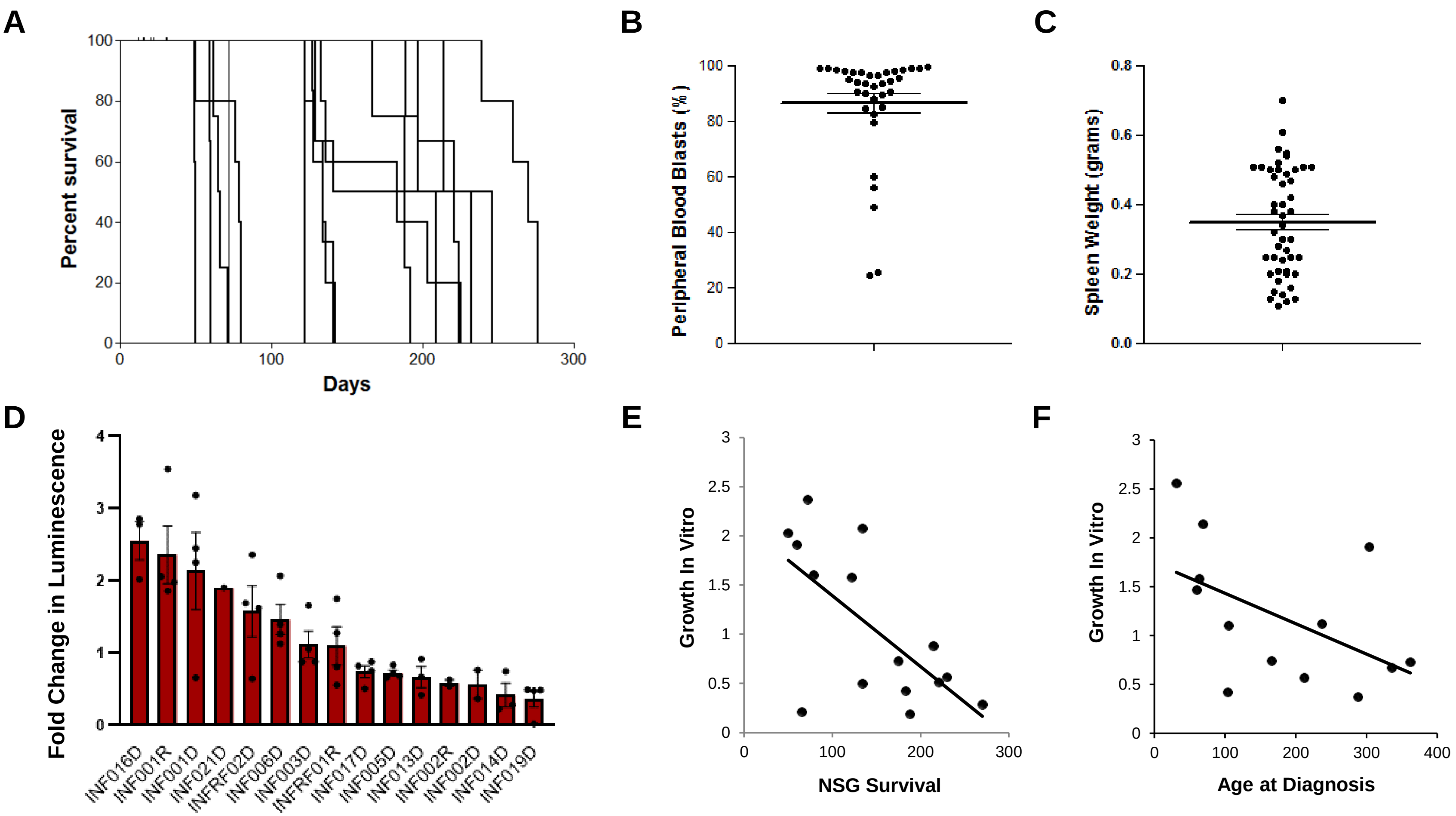

Supplementary Figure 1. Platform to Identify Active Agents in Infantile ALL. (A) Survival of xenografted mice. Five immunodeficient mice were injected for each patient specimen (N=15 specimens). Survival curves for mouse cohorts receiving each of the patient specimens are shown. (B) Blast percentage in the peripheral blood of mice at the time of sacrifice as determined by flow cytometry for human CD45. N=35 biologic replicates, mean and standard error of the mean are shown. (C) Weight of spleen at the time of sacrifice for all mice. N=47 biologic replicates, mean and standard error of the mean are shown (D) In vitro growth at 72 hours for patient specimens. D indicates samples obtained at diagnosis, R indicates relapsed specimens. Cells were thawed and plated overnight in media. The following morning baseline luminescence was measured (t=0) and then again after 72 hours (t=72) in the absence of drug. Fold change was calculated as (t=72)/(t=0). Replicates for each patient sample are cells derived from a single mouse PDX, the mean and the standard error of the mean are shown. (E) Correlation of growth in vitro with median survival of xenografted mice per patient sample. R^2=0.44. (F) Correlation of growth in vitro with age at diagnosis of patients whose leukemia cells were used in the study. R^2=0.27.

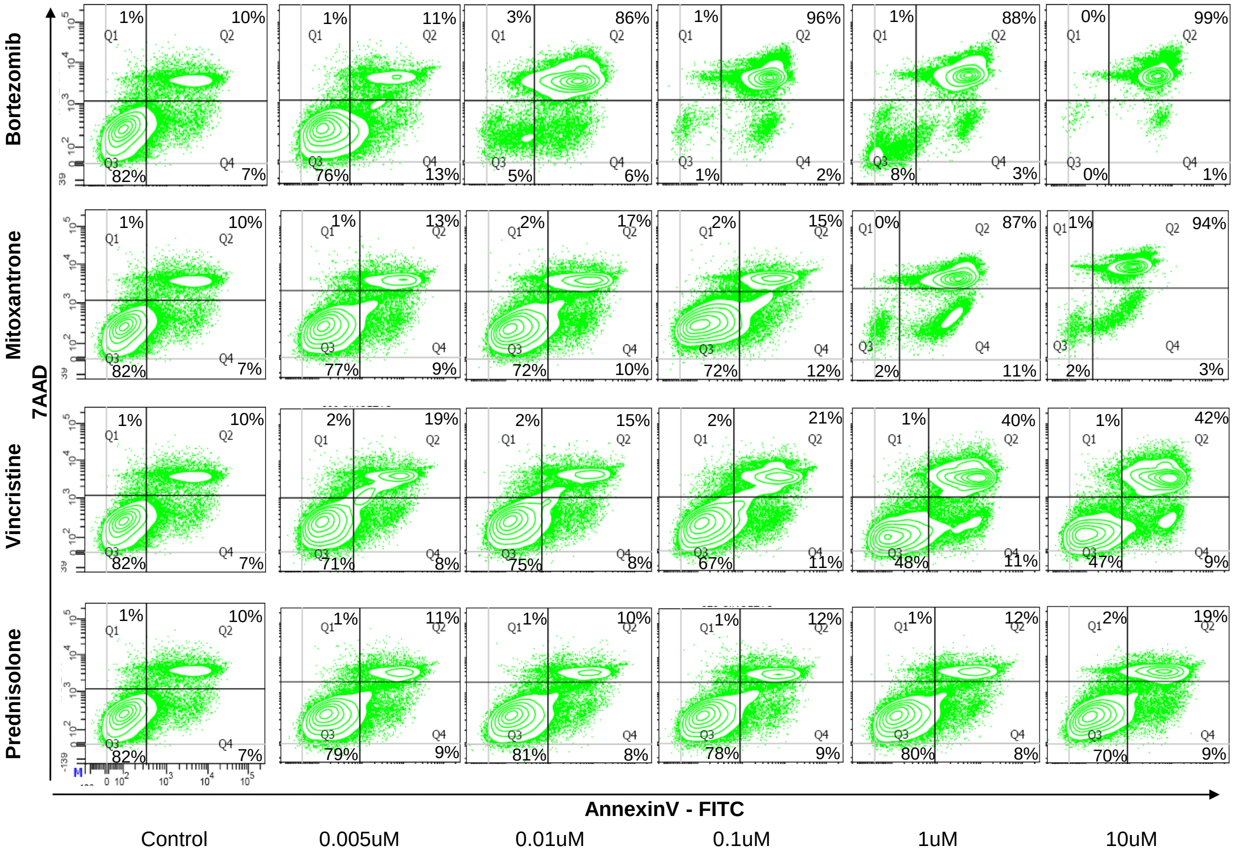

**INF001D IC$_{50}$ Values**		
Drug	XTT	Cell Titer Glo
Bortezomib	0.003890 µM	0.005792 µM
Mitoxantrone	0.1297 µM	0.1595 µM
Vincristine	≥9.7 µM	≥9.7 µM
Prednisolone	≥9.7 µM	≥9.7 µM

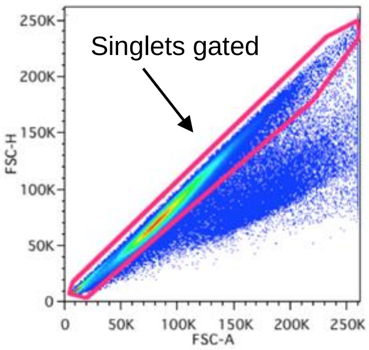

Supplementary Figure 2. Cell titer glo as a surrogate for apoptosis.
Patient sample INF001D was treated with single agent bortezomib, mitoxantrone, vincristine, and prednisolone at indicated concentrations and assayed at 72 hours for viability as determined by flow-based apoptosis, XTT, and Cell Titer Glo as described in methods. Singlets were identified by forward scatter height versus forward scatter area density plot for doublet exclusion as shown next to the table. Experiment was done in duplicate with similar results, data from one experiment is shown.

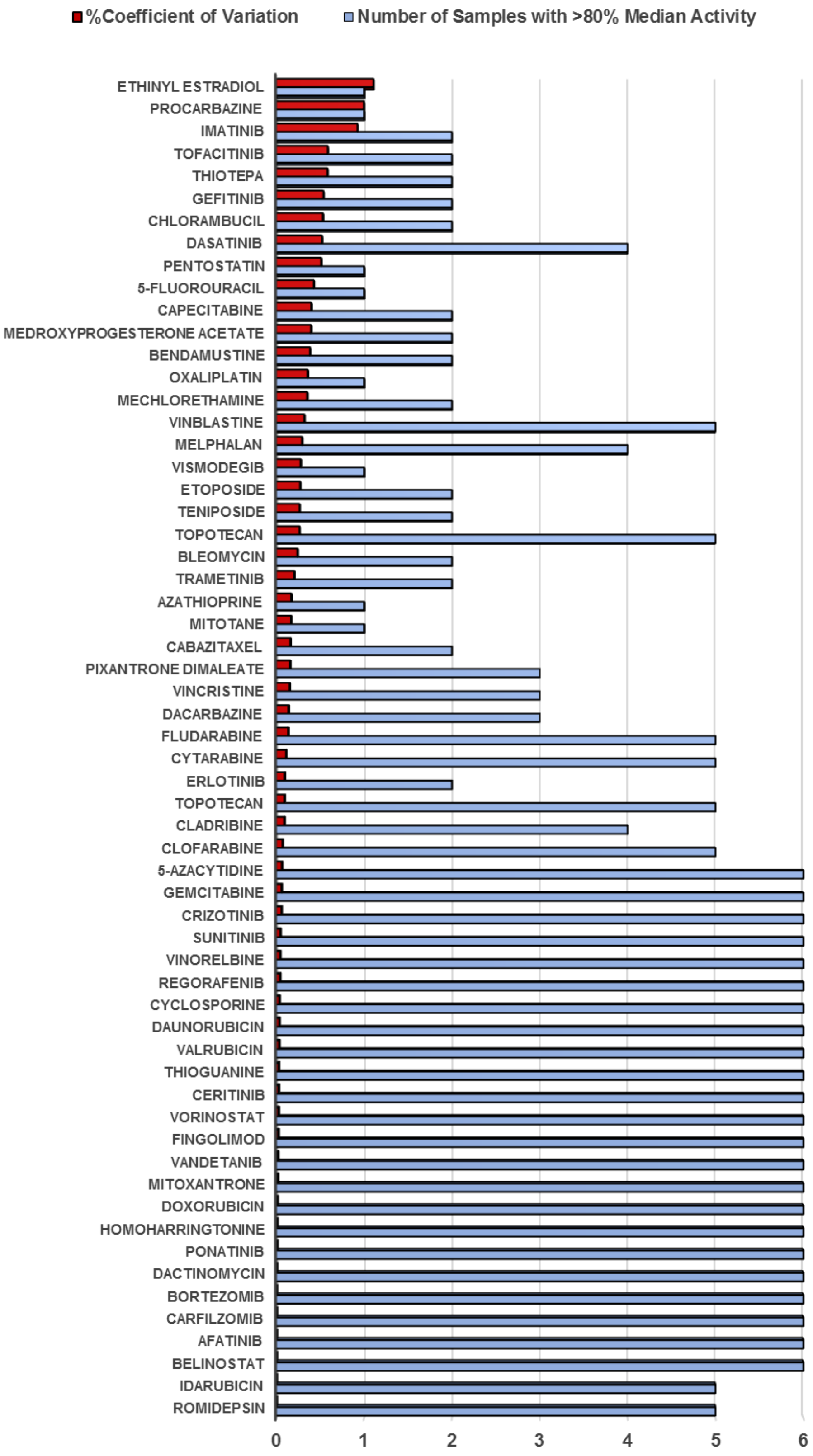

Supplementary Figure 3. Active Antineoplastic Agents. Antineoplastic drugs with >80% activity in one or more patient samples at 10µM. Coefficient of variation (red bars) and number of samples in which the drug was active (blue bars) are shown.

A

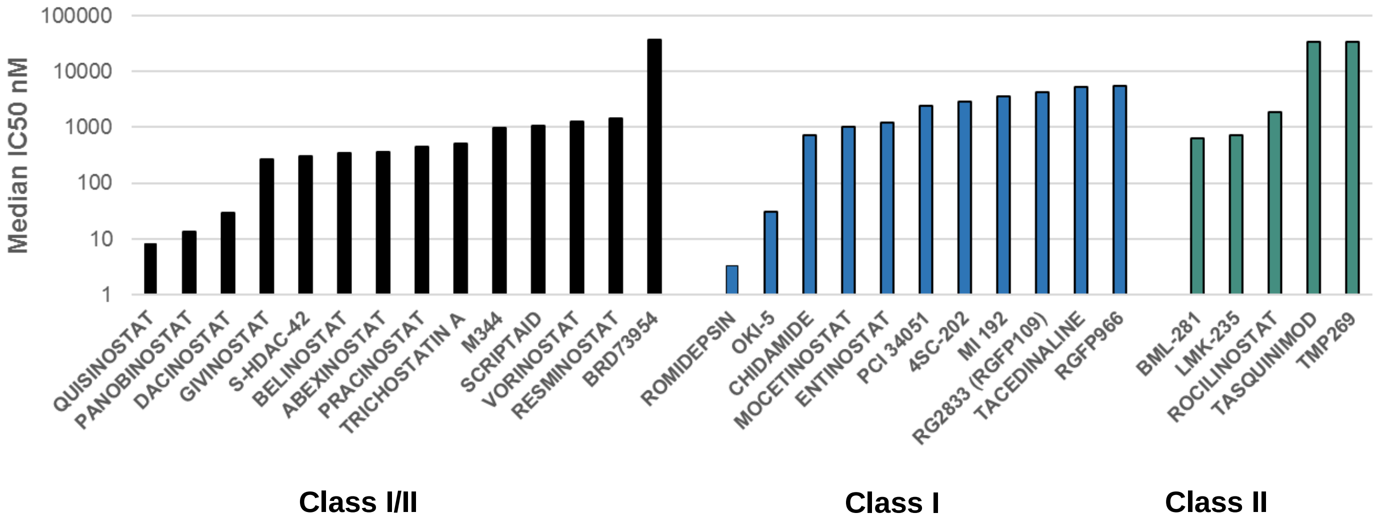

B

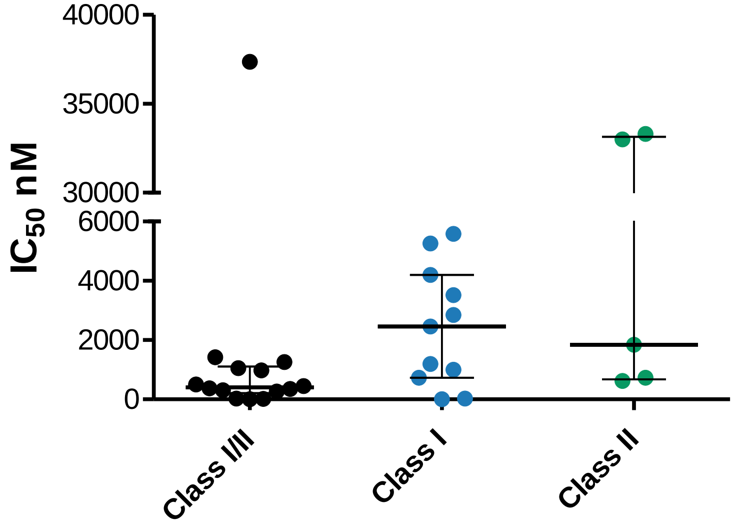

Supplementary Figure 4. Activity of HDAC Inhibitors. A panel of 30 HDAC inhibitors including Class I/II pan inhibitors, class I specific inhibitors, and class II specific inhibitors were evaluated in six patient samples. (A) Median IC_{50} for each of the thirty compounds. (B) Median IC_{50} for each compound as determined in six patient samples was plotted. Median with interquartile range is shown for each class of inhibitors. Class I/II: N=14 compounds each evaluated in six patient samples; Class I: N=11 compounds each evaluated in six patient samples; Class II: N=5 compounds each evaluated in six patient samples.

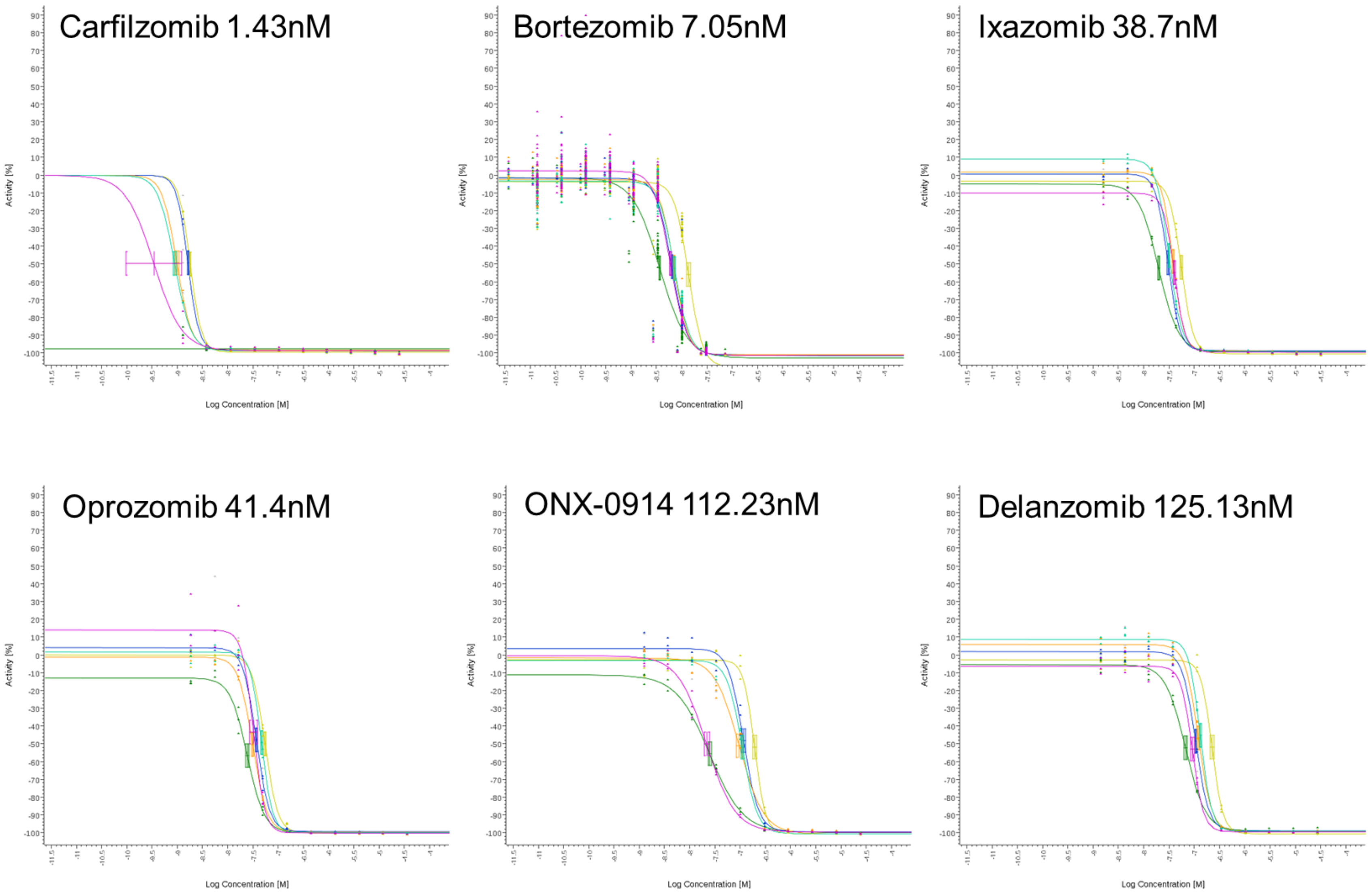

Supplementary Figure 5. Activity of Proteasome Inhibitors in Infant ALL. FDA approved and non-approved proteasome inhibitors were evaluated in six infant ALL specimens with a 10-point dose response assay. Each dose was evaluated in triplicates for each of the six patient samples. Data are presented as a best fit dose response curve. Brackets indicate the confidence interval for the IC_{50} value of each patient sample. IC_{50} curves for all six samples are shown, median IC_{50} for each compound across all six patient samples is indicated above.

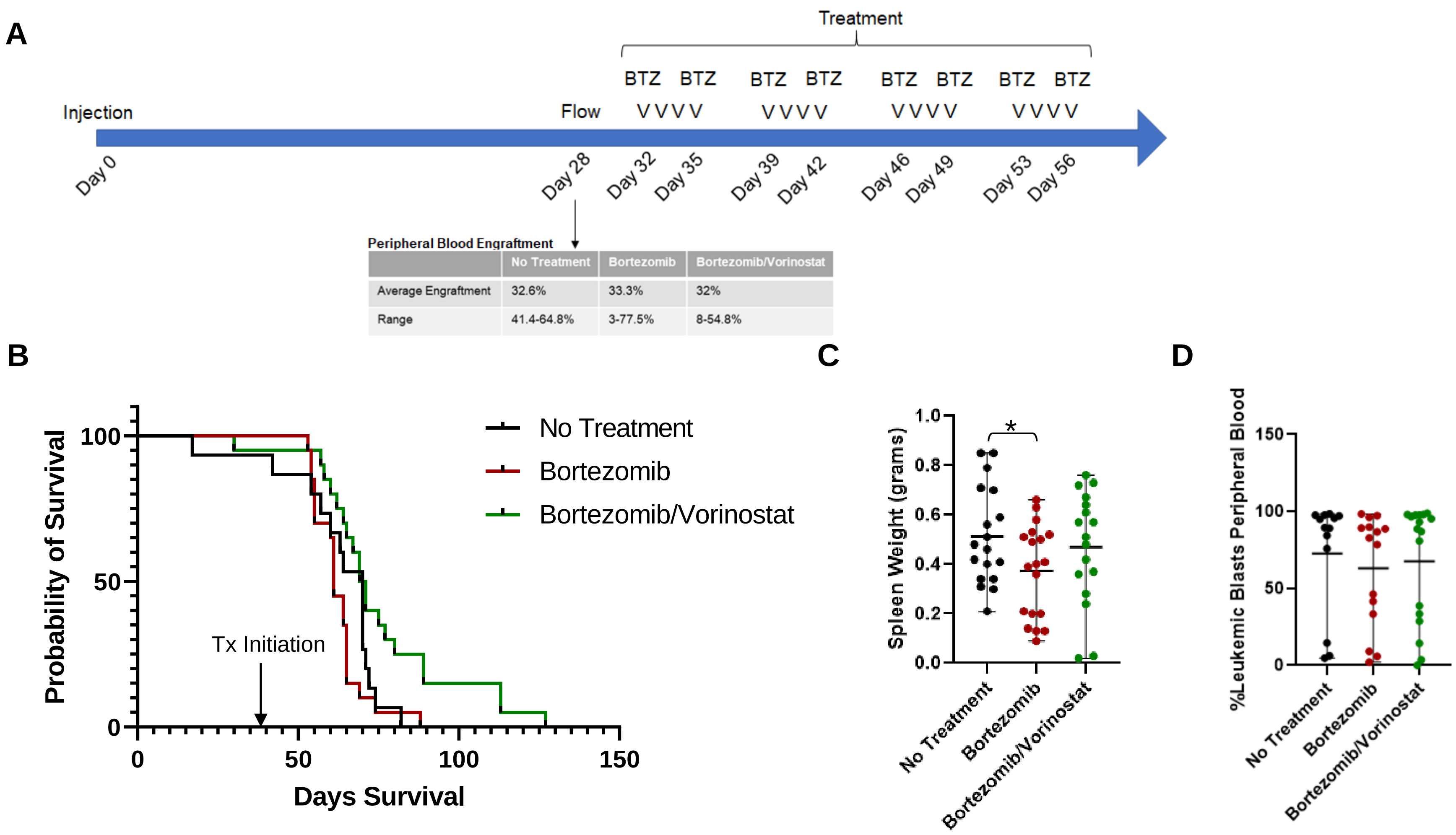

Supplementary Figure 6. Activity of Bortezomib in KMT2Ar Patient Derived Xenograft.

(A) Experimental schema. Mice received one million cells from patient INF001D on day 0. Engraftment was verified on day 28 by flow cytometry of the peripheral blood. Average engraftment and the range are indicated for each cohort. Control mice received PBS injections, while bortezomib treated mice received 0.1 mg/kg/dose intravenously (BTZ) twice weekly beginning on day 32. Vorinostat (V) was administered by oral gavage once daily at 100mg/kg/dose on days 32-35, 39-42, 46-49, and 53-56. (B) Survival of mice. Outcomes of mice were statistically significant *p=0.005* as determined by the Mantel-Cox Log rank test (chi square 10.5, df 2). (C) Disease burden as indicated by spleen weight, p=0.033 Bortezomib vs. No Treatment as determined by unpaired two-tailed t test. There was no difference between Bortezomib/Vorinostat and No Treatment. (D) % Leukemia blasts at the time of sacrifice, one-way ANOVA across all groups *p=0.79,* one-way ANOVA with multiple comparisons was also not significant.

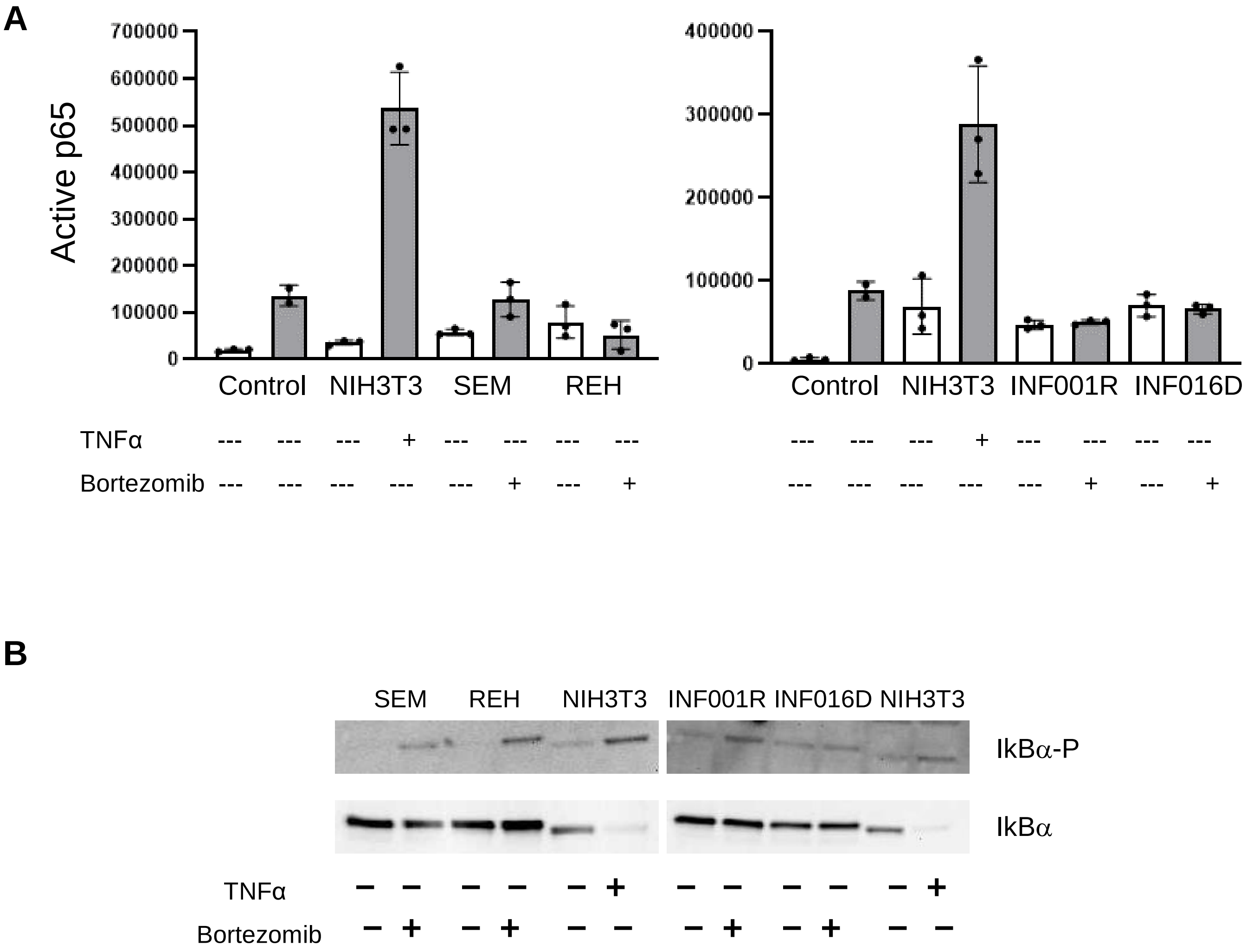

Supplementary Figure 7. NFkB Activity in KMT2Ar Leukemia Cells.
An KMT2Ar infant ALL cell line (SEM), a non-KMT2Ar ALL cell line (REH) and KMT2Ar infant primary patient samples (INF001R and INF016D) were grown in the presence or absence of bortezomib as described in the methods. (A) Active NFkB transcriptional complex was assessed by ELISA as described in the methods. As a positive control, the TNFα treated murine fibroblast cell line NIH3T3 was included. (B) Cells were assessed for total IkBα and phosphorylated IkBα (IkBα-P) by western blot as described in the methods.

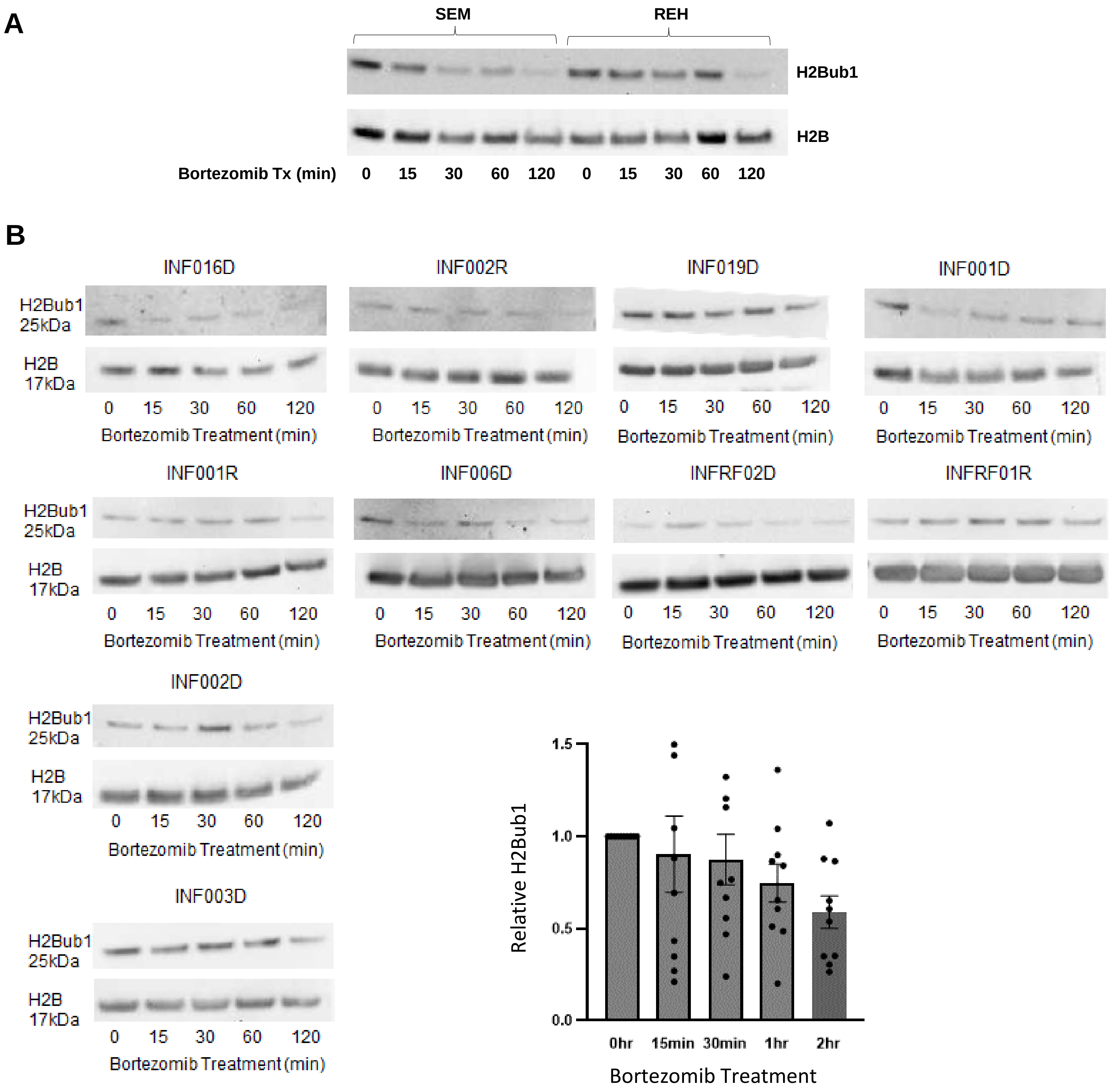

Supplementary Figure 8. Bortezomib Exposure Depletes H2Bub1. (A) Two ALL cell lines were exposed to 50nM of bortezomib for up to 120 minutes. At the indicated time points histones were extracted and blotted for total H2B and H2Bub1 as described in the materials and methods. Shown are representative blots from one experiment which was repeated twice with similar results. (B) Primary patient specimens were exposed to 5nM of bortezomib for up to 120 minutes. At the indicated time points histones were extracted and blotted for total H2B and H2Bub1 as described in the materials and methods. Ten patient specimens are shown. Quantitative summary across all patient specimens evaluated at each time point is shown (average values with standard error of the mean). To determine relative H2Bub1 over time in Figure 3A, the H2Bub1 to total H2B ratio was determined by densitometry at each time point. The ratio at time point zero was set at one and relative values were then determined for subsequent time points. Data are presented as mean values with the standard error of the mean.

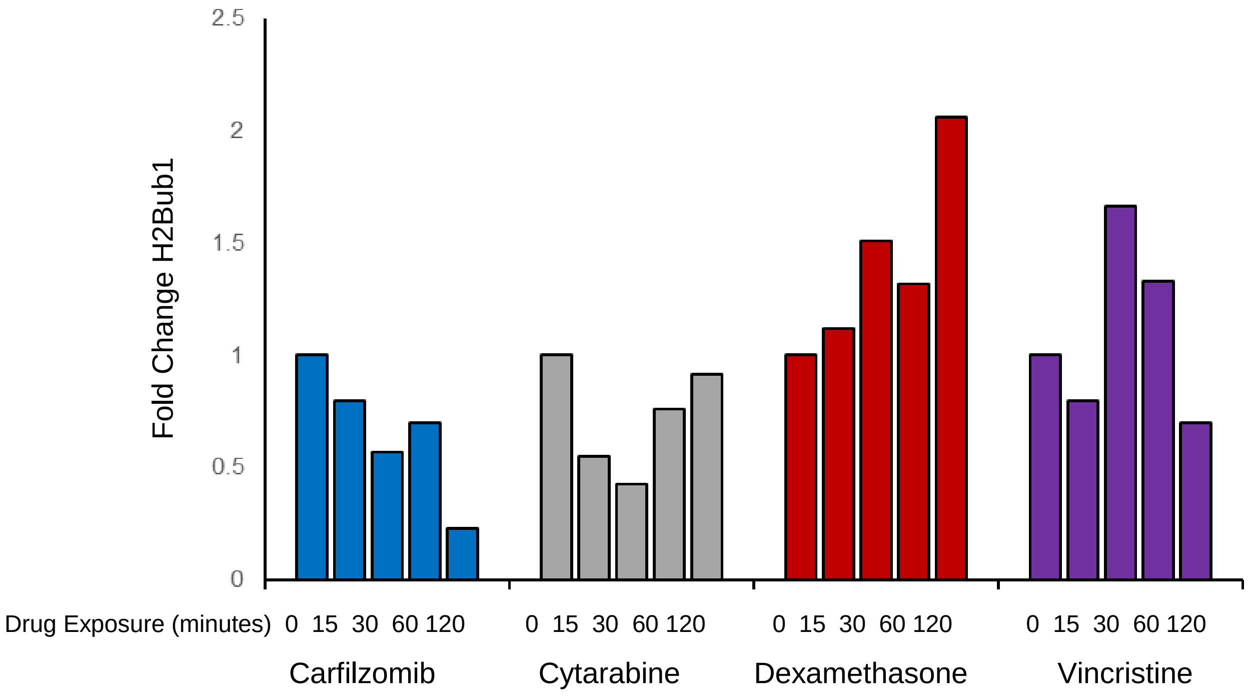

Supplementary Figure 9. H2Bub1 of KMT2Ar Leukemia Following Exposure to Chemotherapy. The KMT2Ar SEM cell line was exposed to 50nM of cytarabine, 500nM vincristine, 500nM dexamethasone, and 10nM carfilzomib for up to 120 minutes. At the indicated time points histones were extracted and blotted for total H2B and H2Bub1 as described in the materials and methods. Two independent experiments were done with similar results, data shown are from one representative experiment. H2Bub1 was normalized to H2B and the ratio was set to one at time 0. Fold change across the time course is shown.

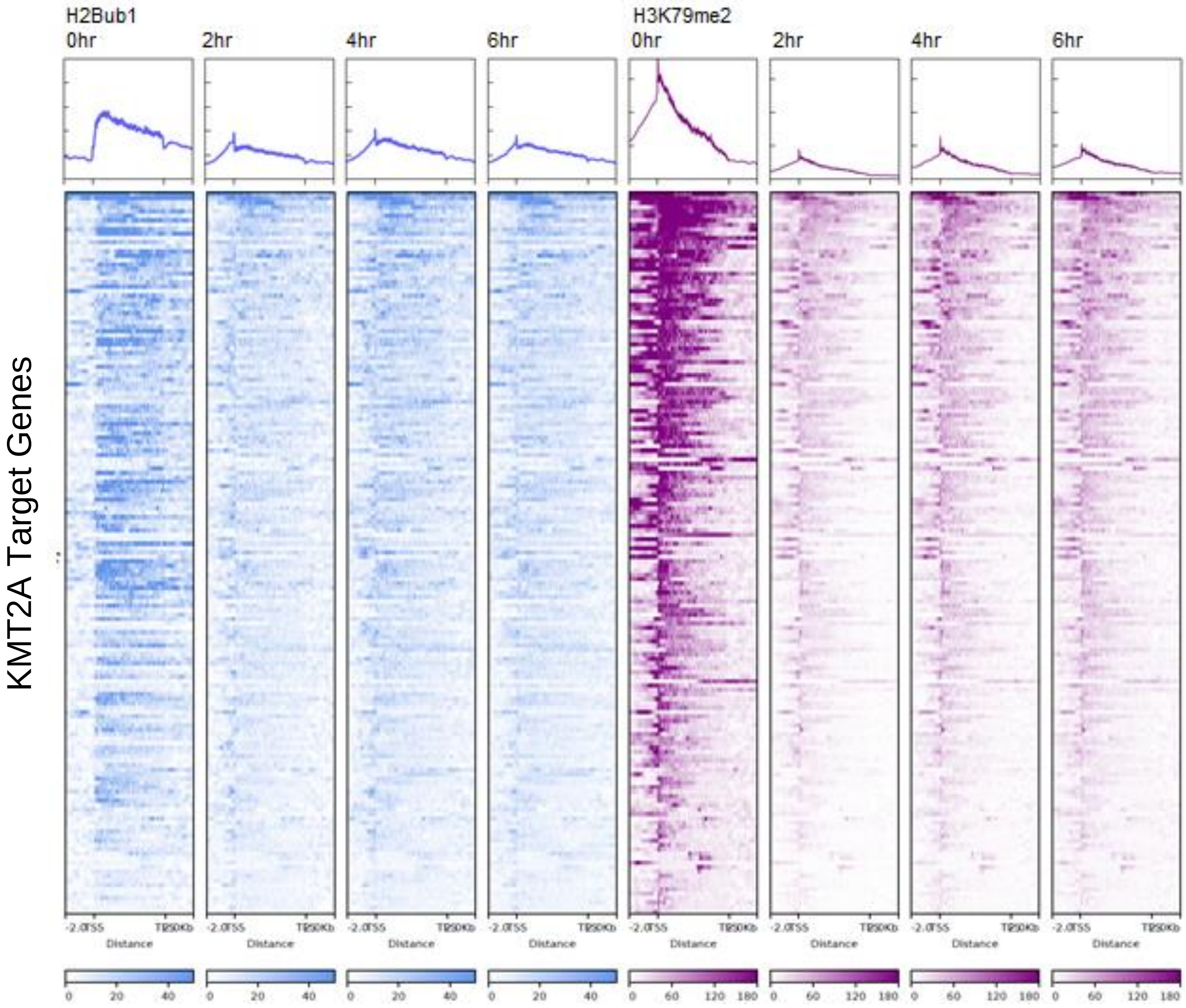

Supplementary Figure 10. Bortezomib Exposure Depletes H2Bub1 and H3K79me2 at KMT2A Target Genes. The SEM cell line was exposed to 50nM of bortezomib for up to 6 hours. To assess DNA associated histones, quantitative ChIP sequencing was done every two hours for H2Bub1 and H3K79me2. Genome wide data are shown in the main manuscript (Figure 3C). KMT2A-AFF1 target genes as determined by ChIP-seq and previously published are shown in this heatmap[4].

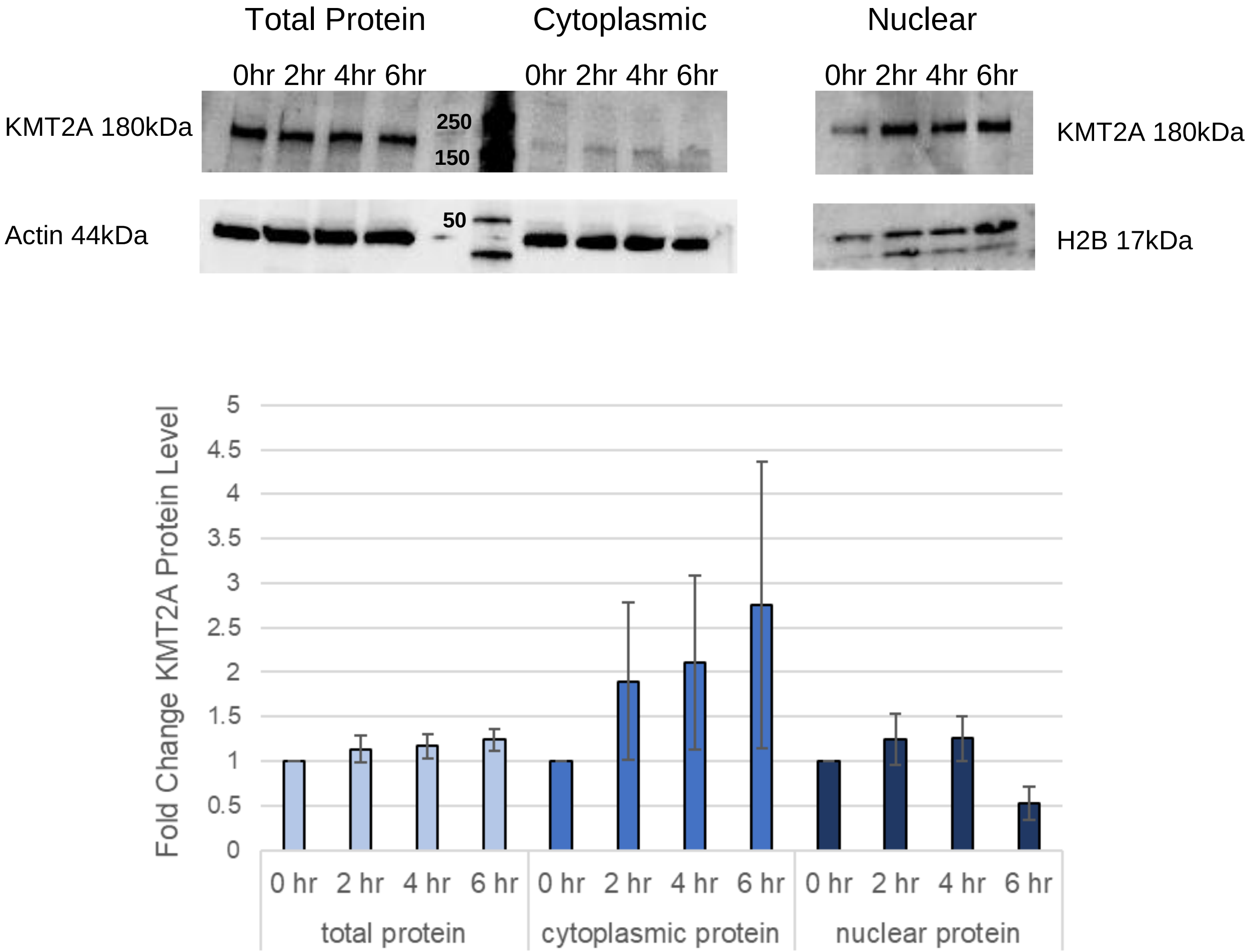

Supplementary Figure 11. Wild type KMT2A Levels Following Proteasome Inhibition. A KMT2Ar infant ALL cell line (SEM) was grown in the presence or absence of bortezomib as described in supplementary methods. Total, cytosolic and nuclear proteins were extracted and blotted for KMT2A, actin, and H2B. Densitometry plots are pooled from three independent experiments, a representative western blot from one of the experiments is shown. KMT2A was normalized to actin for total and cytoplasmic protein extracts. KMT2A was normalized to H2B for the nuclear extract. Data are presented as mean values and the standard error of the mean.

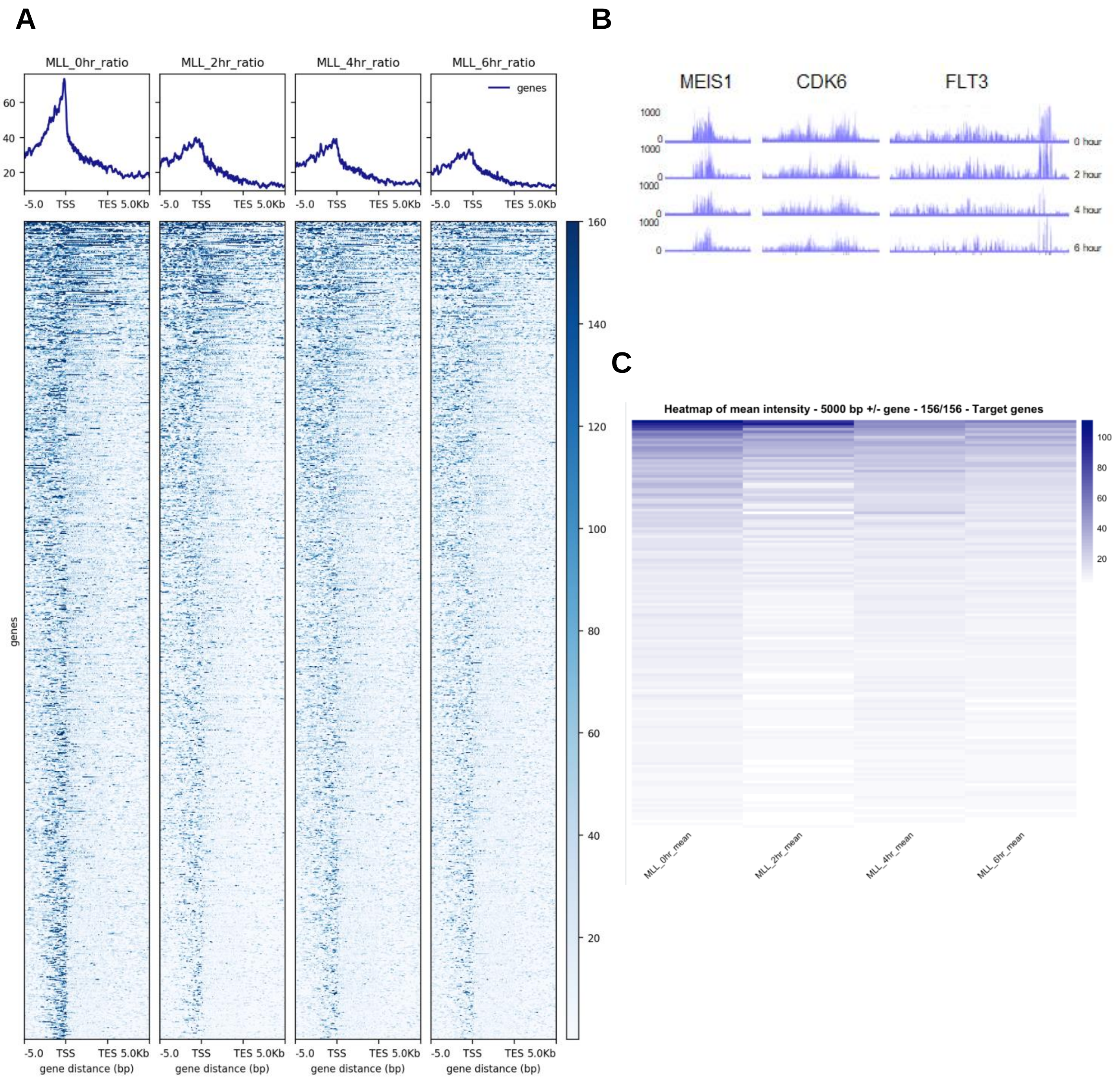

Supplementary Figure 12. Bortezomib Exposure Depletes KMT2A at Target Genes. The SEM cell line was exposed to 50nM of bortezomib for up to 6 hours. To assess DNA associated with KMT2A, CUT&RUN was done every two hours for KMT2A using an N-terminal antibody. (A) Genome wide data (B) Profiles of three KMT2A-AFF1 target genes (C) KMT2A-AFF1 target genes as determined by ChIP-seq and previously published are shown in this heatmap[4].

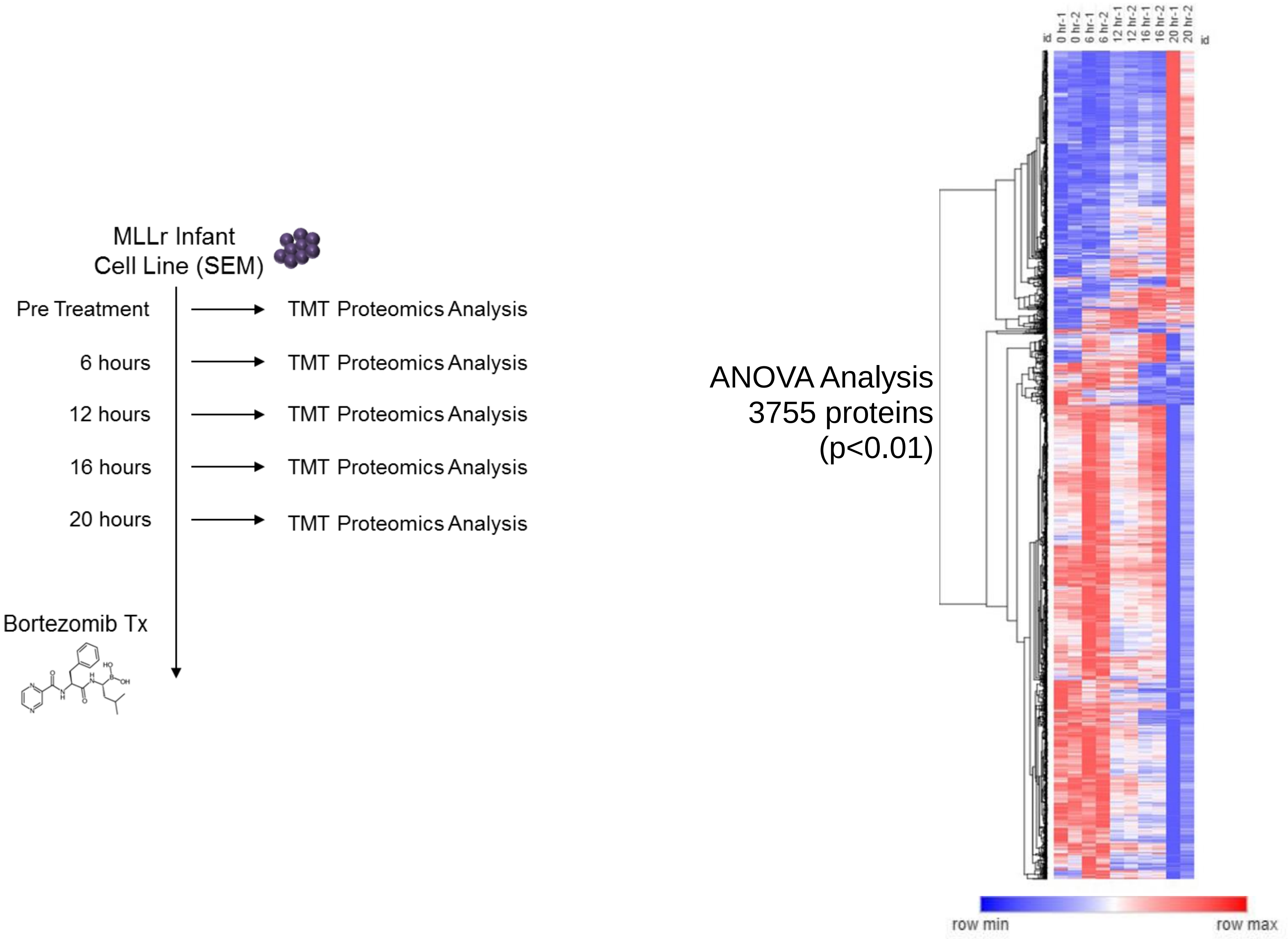

Supplementary Figure 13. Global Protein Changes of KMT2r Leukemia Following Proteasome Inhibition.
The KMT2Ar infant ALL cell line SEM was grown in the presence of bortezomib at 50nM. Samples underwent TMT proteomics analysis as described in materials and methods at 6, 12, 16, and 20 hours in duplicate. 9514 proteins were quantified. See Supplementary Data 7 for proteins, signal change, p and FDR values.

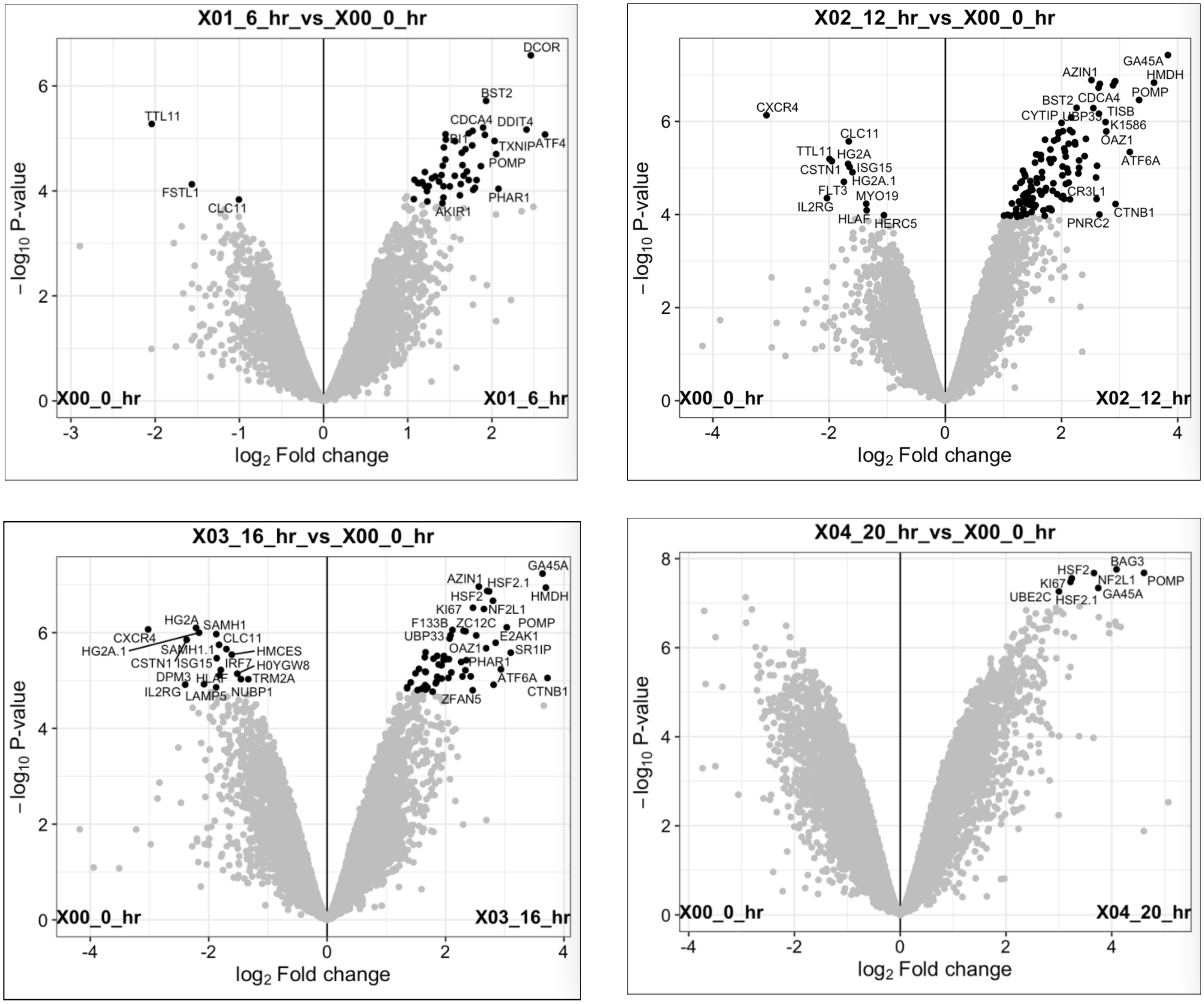

Supplementary Figure 14. Differentially Expressed Proteins of KMT2Ar Leukemia Following Proteasome Inhibition.

The KMT2Ar infant ALL cell line SEM was exposed to 50nM bortezomib and samples underwent TMT proteomics analysis as described in materials and methods at 0, 6, 12, 16, and 20 hours in duplicate. Differential Expression of the Proteome was determined by the DEP pipeline by applying empirical Bayes statistics on protein-wise linear models using limma as previously described [5]. Volcano plots at each time point compared to baseline are shown. See also Supplementary Data 8.

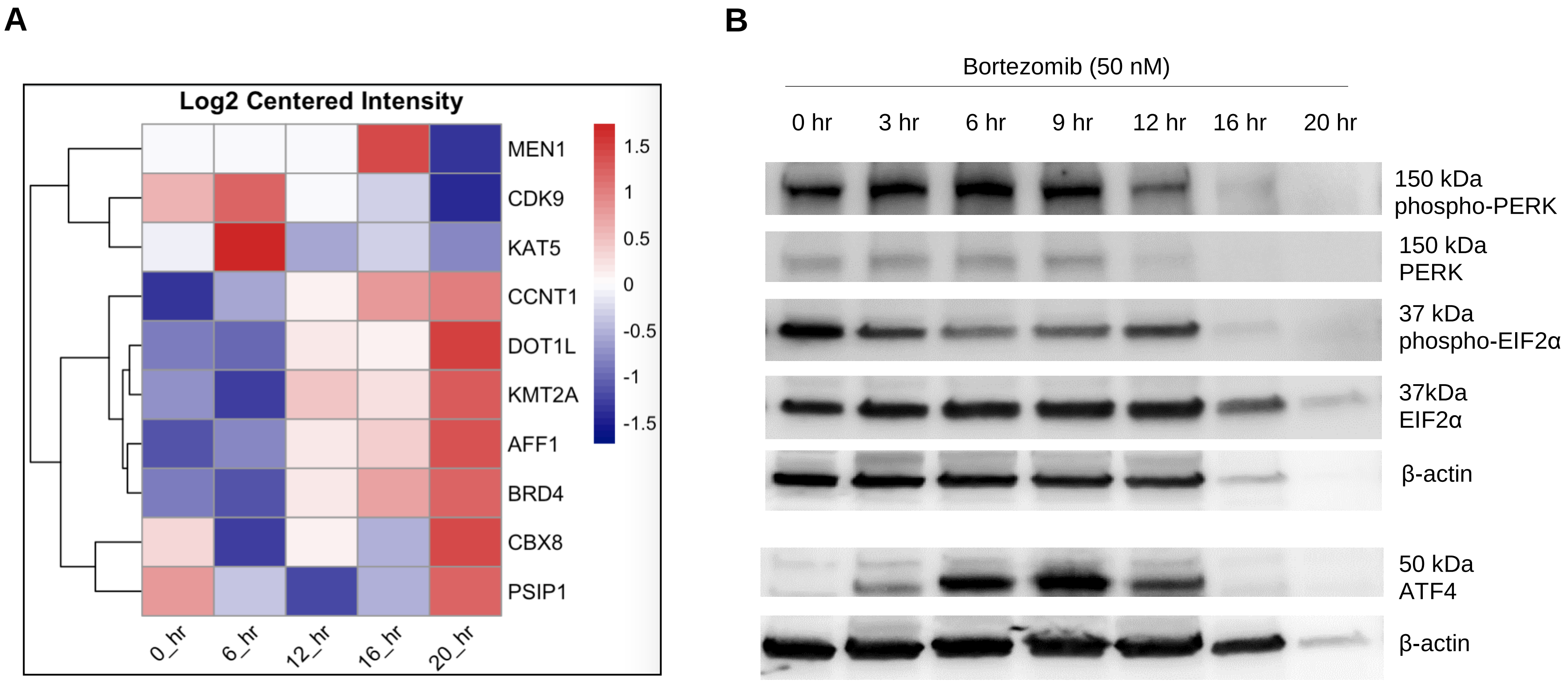

Supplementary Figure 15. KMT2Ar Complex Accumulation and Proteotoxic Stress in Bortezomib Treated Cells.
(A) The KMT2Ar infant ALL cell line SEM was exposed to 50nM bortezomib and samples underwent TMT proteomics analysis as described in materials and methods at 0, 6, 12, 16, and 20 hours in duplicate. Log2 centered intensity following DEP analysis is shown[5]. (B) The KMT2Ar infant ALL cell line SEM cell cycle was synchronized by exposure to 250 nM PD0332991 (palbociclib) for 24 hours. PD0332991 was then washed out and cells were placed in 50nM bortezomib for the indicated incubation periods. Total proteins were extracted for western blot analysis. Shown are representative blots from one experiment. The experiment was repeated three times with similar results.

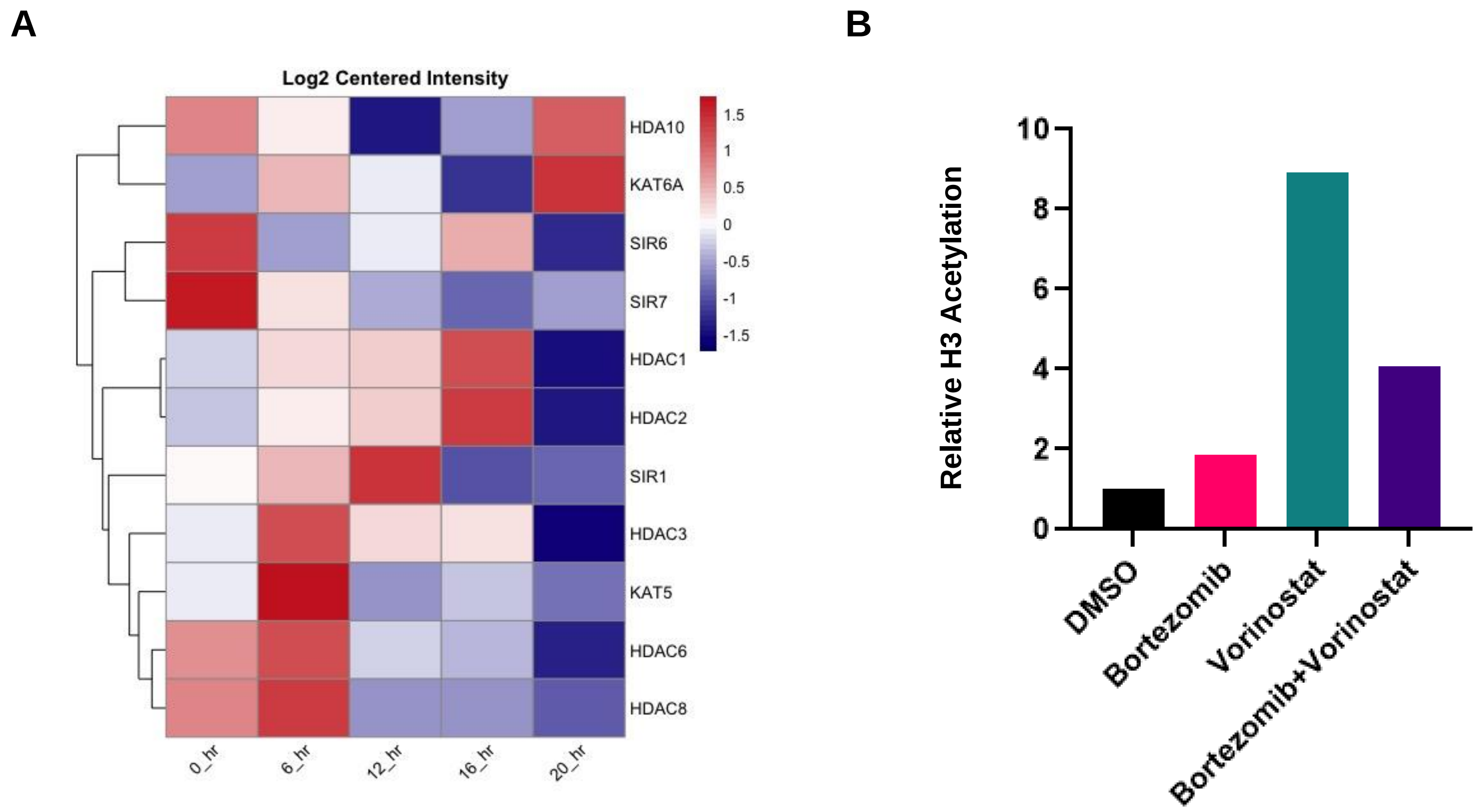

Supplementary Figure 16. Histone Acetylation in Bortezomib and Vorinostat Treated Cells.
(A) The KMT2Ar infant ALL cell line SEM was exposed to 50nM bortezomib and samples underwent TMT proteomics analysis as described in materials and methods at 0, 6, 12, 16, and 20 hours in duplicate. Log2 centered intensity of HDACs and HATs following DEP analysis is shown[5]. (B) SEM cells were incubated for 20 hours in DMSO, 5nM bortezomib, 1uM vorinostat, or both. Total protein was extracted as described in materials and methods, blotted, and stained for acetylated histone H3 and total H3. Densitometry of acetylated histone H3 relative to total H3 is shown from one representative experiment. Two independent experiments were done with similar results.

SEM Cell Line | INF006D | INF001D

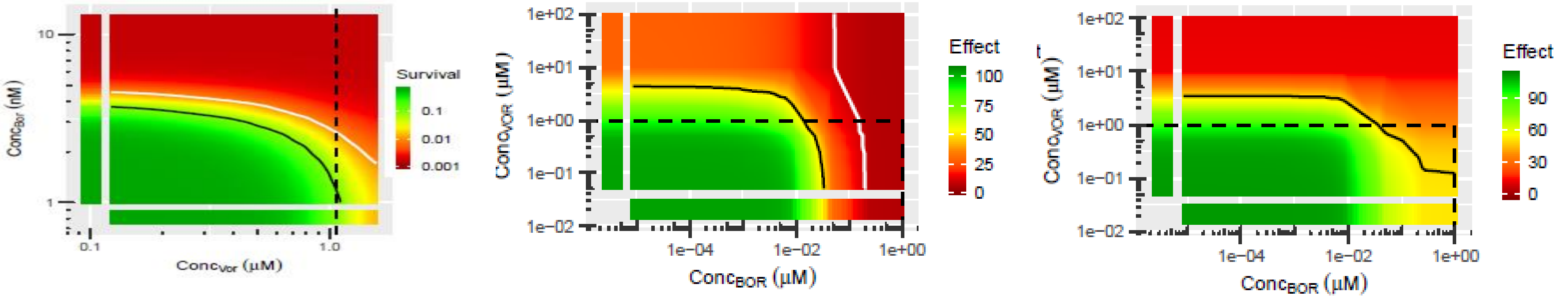

K=-0.542-0.351 | K=-0.158-0.436 | K=-0.347-0.124

Supplementary Figure 17. Combinatorial Treatment of KMT2Ar Leukemia with Bortezomib and Vorinostat.
Cytotoxicity assay of bortezomib in combination with vorinostat. The KMT2Ar infant ALL cell line SEM and two patient samples were grown in the presence of bortezomib and vorinostat at the indicated concentrations for 72 hours. Viability was assessed by cell titer glo. Bivariate Response to Additive Interacting Doses (BRAID), an analysis of fitted response surfaces was done to evaluate the interaction. The kappa confidence intervals are shown which center around zero and are consistent with an additive effect in all three specimens[6].

.

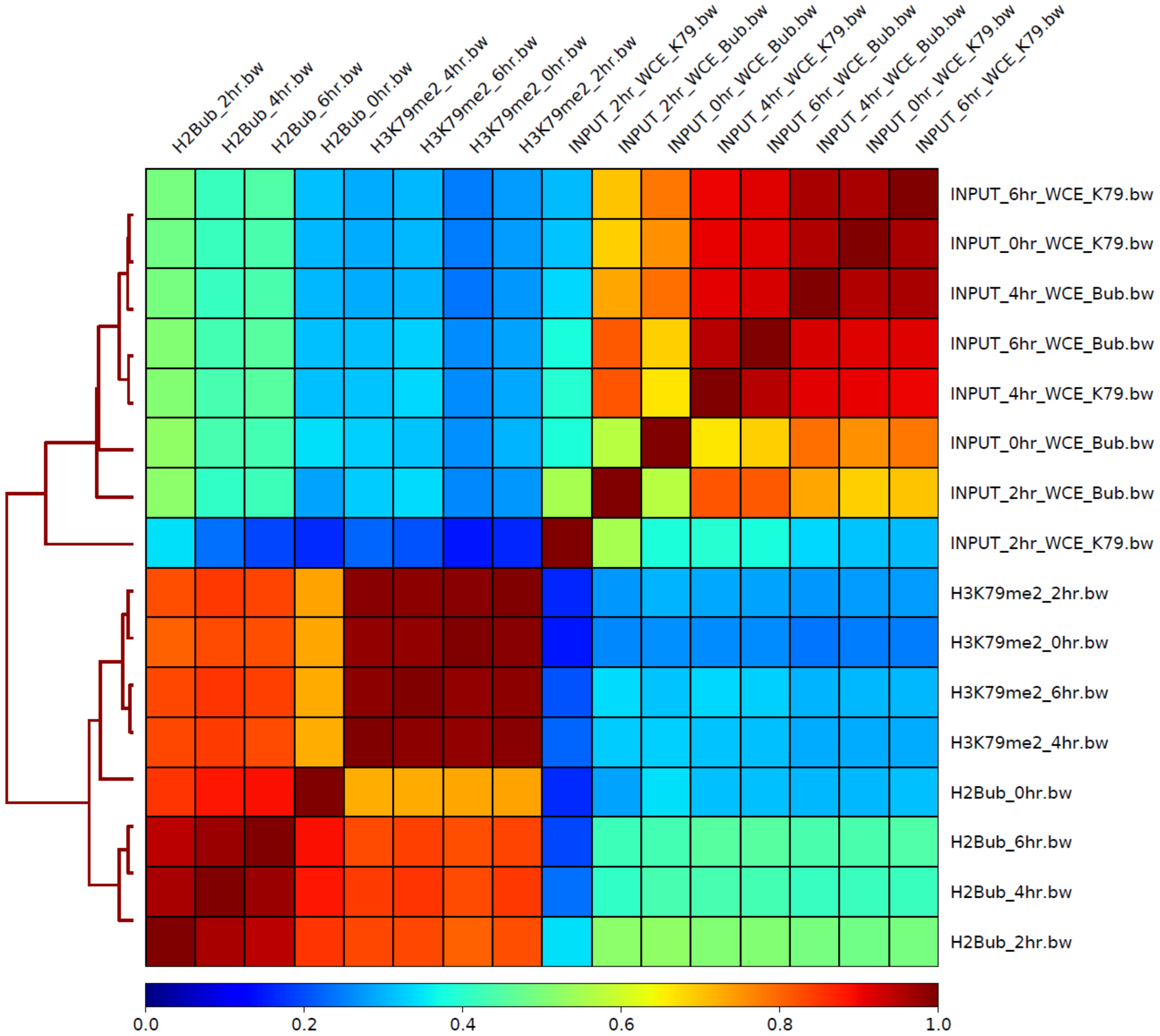

Supplementary Figure 18. ChIP-Rx Correlation Plots.
Correlation plots by multiBigwigSummary from deeptools (v3.0.2-1-ac19361) of ChIP-Rx.

Supplementary References

1. Styczynski J*, et al.* Comparison of clofarabine activity in childhood and adult acute leukemia: individual tumor response study. *Anticancer research* **29**, 1643-1650 (2009).

2. Szczepanek J*, et al.* Differential ex vivo activity of bortezomib in newly diagnosed paediatric acute lymphoblastic and myeloblastic leukaemia. *Anticancer research* **30**, 2119-2124 (2010).

3. Keshelava N*, et al.* Initial testing (stage 1) of vorinostat (SAHA) by the pediatric preclinical testing program. *Pediatric blood & cancer* **53**, 505-508 (2009).

4. Guenther MG*, et al.* Aberrant chromatin at genes encoding stem cell regulators in human mixed-lineage leukemia. *Genes & development* **22**, 3403-3408 (2008).

5. Zhang X, Smits AH, van Tilburg GB, Ovaa H, Huber W, Vermeulen M. Proteome-wide identification of ubiquitin interactions using UbIA-MS. *Nature protocols* **13**, 530-550 (2018).

6. Twarog NR, Martinez NE, Gartrell J, Xie J, Tinkle CL, Shelat AA. Using response surface models to analyze drug combinations. *Drug Discov Today* **26**, 2014-2024 (2021).
