# Supplementary material for: Proteasome inhibition targets the KMT2A transcriptional complex in acute lymphoblastic leukemia
Source: Nat Commun. 2023 Feb 13;14:809. doi: 10.1038/s41467-023-36370-x (PMC9925443; doi:10.1038/s41467-023-36370-x)
Supplement: Supplementary file 2 — Description of Additional Supplementary Files [file 41467_2023_36370_MOESM2_ESM.docx]

**Description of Additional Supplementary Files**

Supplementary Data 1

Description: FDA Approved Compounds Used in Screening

Supplementary Data 2

Description: Activity of FDA Approved Compounds at 10uM in Infant ALL

Supplementary Data 3

Description: Secondary Validation and IC50 Determination of Top Hits

Supplementary Data 4

Description: Peak Calls KMT2A Targets ChIP-Rx SEM Cells

Supplementary Data 5

Description: Differentially Expressed Genes in Bortezomib Treated Patient Samples

Supplementary Data 6

Description: TMT Proteome Profiling

Supplementary Data 7

Description: ANOVA Analysis Proteome Time Course

Supplementary Data 8

Description: Differential Enrichment Proteome Analysis of Bortezomib Treated KMT2Ar Leukemia Cells

Supplementary Data 9

Description: Enriched Hallmark Pathways at 6 Hours Following Exposure to Proteasome Inhibition in KMT2Ar Leukemia

Supplementary Data 10

Description: Enriched Hallmark Pathways at 12 Hours Following Exposure to Proteasome Inhibition in KMT2Ar Leukemia

Supplementary Data 11

Description: Enriched Hallmark Pathways at 16 Hours Following Exposure to Proteasome Inhibition in KMT2Ar Leukemia

Supplementary Data 12

Description: Enriched Hallmark Pathways at 20 Hours Following Exposure to Proteasome Inhibition in KMT2Ar Leukemia

Supplementary Data 13

Description: Enriched KEGG Pathways at 6 Hours Following Exposure to Proteasome Inhibition in KMT2Ar Leukemia

Supplementary Data 14

Description: Enriched KEGG Pathways at 12 Hours Following Exposure to Proteasome Inhibition in KMT2Ar Leukemia

Supplementary Data 15

Description: Enriched KEGG Pathways at 16 Hours Following Exposure to Proteasome Inhibition in KMT2Ar Leukemia

Supplementary Data 16

Description: Enriched KEGG Pathways at 20 Hours Following Exposure to Proteasome Inhibition in KMT2Ar Leukemia

Supplementary Data 17

Description: Enriched Reactome Pathways at 6 Hours Following Exposure to Proteasome Inhibition in KMT2Ar Leukemia

Supplementary Data 18

Description: Enriched Reactome Pathways at 12 Hours Following Exposure to Proteasome Inhibition in KMT2Ar Leukemia

Supplementary Data 19

Description: Enriched Reactome Pathways at 16 Hours Following Exposure to Proteasome Inhibition in KMT2Ar Leukemia

Supplementary Data 20

Description: Enriched Reactome Pathways at 20 Hours Following Exposure to Proteasome Inhibition in KMT2Ar Leukemia
