# Supplementary material for: Proteasome inhibition targets the KMT2A transcriptional complex in acute lymphoblastic leukemia
Source: Nat Commun. 2023 Feb 13;14:809. doi: 10.1038/s41467-023-36370-x (PMC9925443; doi:10.1038/s41467-023-36370-x)
Supplement: Supplementary file 4 — Reporting Summary [file 41467_2023_36370_MOESM4_ESM.pdf]

nature portfolio

Corresponding author(s): Tanja A. Gruber

Last updated by author(s): December 28, 2022

Reporting Summary

Nature Portfolio wishes to improve the reproducibility of the work that we publish. This form provides structure for consistency and transparency in reporting. For further information on Nature Portfolio policies, see our Editorial Policies and the Editorial Policy Checklist.

Statistics

For all statistical analyses, confirm that the following items are present in the figure legend, table legend, main text, or Methods section.

n/a	Confirmed	
☐	☒	The exact sample size (*n*) for each experimental group/condition, given as a discrete number and unit of measurement
☐	☒	A statement on whether measurements were taken from distinct samples or whether the same sample was measured repeatedly
☐	☒	The statistical test(s) used AND whether they are one- or two-sided<br>*Only common tests should be described solely by name; describe more complex techniques in the Methods section.*
☒	☐	A description of all covariates tested
☒	☐	A description of any assumptions or corrections, such as tests of normality and adjustment for multiple comparisons
☐	☒	A full description of the statistical parameters including central tendency (e.g. means) or other basic estimates (e.g. regression coefficient) AND variation (e.g. standard deviation) or associated estimates of uncertainty (e.g. confidence intervals)
☐	☒	For null hypothesis testing, the test statistic (e.g. *F*, *t*, *r*) with confidence intervals, effect sizes, degrees of freedom and *P* value noted<br>*Give P values as exact values whenever suitable.*
☒	☐	For Bayesian analysis, information on the choice of priors and Markov chain Monte Carlo settings
☒	☐	For hierarchical and complex designs, identification of the appropriate level for tests and full reporting of outcomes
☐	☒	Estimates of effect sizes (e.g. Cohen's *d*, Pearson's *r*), indicating how they were calculated

Our web collection on statistics for biologists contains articles on many of the points above.

Software and code

Policy information about availability of computer code

Data collection	No software used
Data analysis	Published analysis tools as described in methods. Genedata Screener v15.0, ggplot2 v3.4.0, Limma R package v3.16, JUMP software suite v1.0.55, samtools v1.16.1, deeptools v3.0.2-1-ac19361, voom packge (R 3.23, edgeR 3.12.1, limma 3.26.9), FlowJo v9

For manuscripts utilizing custom algorithms or software that are central to the research but not yet described in published literature, software must be made available to editors and reviewers. We strongly encourage code deposition in a community repository (e.g. GitHub). See the Nature Portfolio guidelines for submitting code & software for further information.

Data

Policy information about availability of data

All manuscripts must include a data availability statement. This statement should provide the following information, where applicable:

- Accession codes, unique identifiers, or web links for publicly available datasets
- A description of any restrictions on data availability
- For clinical datasets or third party data, please ensure that the statement adheres to our policy

ChIP sequencing and gene expression data are available in the GEO repository: https://www.ncbi.nlm.nih.gov/geo, GSE152816. Results from proteome profiling by TMT are included in the supplementary materials and have also been deposited to the ProteomeXchange Consortium via the Proteomics IDEntifications Database (PRIDE) partner repository with the dataset identifier PXD038845: https://www.ebi.ac.uk/pride67, 68. Correspondence and material requests should be addressed to Tanja A. Gruber: tagruber@stanford.edu

Human research participants

Policy information about studies involving human research participants and Sex and Gender in Research.

Reporting on sex and gender	Patient sex is included in supplementary table 25
Population characteristics	relapsed/refractory KMT2A rearranged acute lymphoblastic leukemia and acute myeloid leukemia pediatric patients. Age:at the time of treatment: 5 months, 7 months, 8 months, 18 months, 11 years, 3.5 years, 1.5 years, 9 years, 2.3 years, 4 years, and 14 months. Gender/sex: 3 males and 7 females
Recruitment	Patients receiving care at St. Jude Children's Research Hospital who were multiply relapsed or refractory to treatment were offered salvage regimens on a compassionate use basis
Ethics oversight	Pediatric patients with relapsed/refractory KMT2Ar leukemia who were not eligible for a clinical trial received salvage chemotherapy regimens containing bortezomib, an anthracycline and vorinostat as previously published for hematologic malignancies on a compassionate basis. Cases were reviewed following IRB approval from St. Jude Children's Research Hospital. Written informed consent for chemotherapy was obtained from the parents or guardians at the time of treatment according to CARE guidelines and in compliance with the Declaration of Helsinki principles.

Note that full information on the approval of the study protocol must also be provided in the manuscript.

Field-specific reporting

Please select the one below that is the best fit for your research. If you are not sure, read the appropriate sections before making your selection.

☒ Life sciences ☐ Behavioural & social sciences ☐ Ecological, evolutionary & environmental sciences

For a reference copy of the document with all sections, see nature.com/documents/nr-reporting-summary-flat.pdf

Life sciences study design

All studies must disclose on these points even when the disclosure is negative.

Sample size	Experiments with patient samples: 6 patient samples were grown successfully in vitro and used for high throughput drug screening. Samples that did not grow in vitro were excluded.<br>Histone Extractions and Analysis (Figure 3A) - all six patient samples that demonstrated growth in vitro were included and four patient samples that did not grow in vitro were included in this experiment to ensure reproducibility across samples with varying growth characteristics.<br>CUT&RUN PCR: Four patient samples that demonstrated growth in vitro for which there was sufficient specimens to meet the minimal input requirement were chosen.<br>RNASeq (Figure 3E)- 5 patient samples that demonstrated growth in vitro were selected for this experiment. Samples were chosen based on sufficient material availability.<br>Combinatorial drugging (Figure 4B): Two patient samples that demonstrated growth in vitro and had sufficient material availability for combinatorial drugging were selected in addition to a cell line (SEM) that had the required characteristics (infant ALL with KMT2Ar).
Data exclusions	no data exclusions
Replication	Figure 1: Six passaged patient specimens were thawed and cultured in the presence of compounds at a single concentration of 10µM in duplicate<br>Figure 3A: 10 unique patient samples were analyzed for H2Bub1 - 3 samples in expt 1, 3 samples in expt 2, and 4 samples in expt 4.<br>Figure 3B: 4 unique patient samples were analyzed by KMT2A CUT&RUN - 2 samples in expt 1 and 2 samples in expt 2<br>Figure 3C: ChIP-RX was performed twice with similar results. A representative experiment is shown.

	Figure 3E: Experiment was performed twice - the first time with two patient samples using gene expression arrays, the second time with five unique patient samples using RNA sequencing.<br>Figure 3G: Four independent experiments<br>Figure 4A: Two independent experiments<br>Figure 4B: Three experiments with three unique samples (SEM cell line and two patient samples)
Randomization	Murine In Vivo Studies: Mice engrafted with leukemia were treated with DMSO, bortezomib, or bortezomib+vorinostat treatment. Mice were not randomized. Engraftment levels were not statistically different between cohorts.<br>Patients treated with bortezomib and vorinostat containing regimens were not randomized, they received treatment on a compassionate use basis.
Blinding	Blinding of murine in vivo studies and patients treated was not possible as administration of the correct drug was necessary. For murine experiments drug treatments were required to be specified on cage cards as per animal facility requirements.

Reporting for specific materials, systems and methods

We require information from authors about some types of materials, experimental systems and methods used in many studies. Here, indicate whether each material, system or method listed is relevant to your study. If you are not sure if a list item applies to your research, read the appropriate section before selecting a response.

Materials & experimental systems

n/a	Involved in the study
☐	☒ Antibodies
☐	☒ Eukaryotic cell lines
☒	☐ Palaeontology and archaeology
☐	☒ Animals and other organisms
☐	☒ Clinical data
☒	☐ Dual use research of concern

Methods

n/a	Involved in the study
☐	☒ ChIP-seq
☐	☒ Flow cytometry
☒	☐ MRI-based neuroimaging

Antibodies

Antibodies used	BD Biosciences Antibodies for flow cytometry including catalog number, all antibodies used at concentrations as per manufacturers instructions: hCD3 FITC (BD 349201), hCD19 PE (BD 340720), hCD45 PeCy7 (BD 557748), mCD45 APC (BD 553991) Annexin V FITC (BD 560931) 7-AAD (BD 559925)<br>Western Blots: IKBa (cell signaling, cat #4814, 1:1000 dilution), P-IKBa (cell signaling, cat#9246, 1:1000 dilution), anti-H2B (Millipore 07-371; 1:3000 dilution), anti-H2Bub1 (Millipore 05-1312 clone 56; 1:4000 dilution), anti-acetyl Histone H3 (Millipore 07-360, 1:1000 dilution), and anti-H3 (Millipore 04-928, 1:1000 dilution)<br>ChIP Rx: H3K79me2 (12.8µg/ml lysate, Abcam #ab3594), H2B-ub (10µg/ml lysate, Millipore #17-650)<br>CUT&RUN PCR: MLL 0.5ug (Bethyl A300-086A), H2Bub1 0.5ug (Cell Signaling 5546T), and normal rabbit IgG 0.5ug (CUTANATM CUT&RUN kit negative control EpiCypher catalog number 14-1048)<br>Immunofluorescence: 2.0 ug/mL each Anti-acetyl-alpha tubulin (Millipore Sigma, clone 6-11b-1), 1:500 Vimentin Rabbit Monoclonal Antibody (ThermoFisher Scientific clone MA5-16409), Alexa Fluor 555 Goat anti-Rabbit IgG (ThermoFisher Cat#A32732), Alexa Fluor 488 Goat anti-Mouse IgG Secondary Antibody (ThermoFisher Cat#A-11001)
Validation	IKBa: Validated in HeLa, Karpas299, Neuro2A, A20 and C6 cell lines by cell signaling (manufacturer)<br>P-IKBa: Validated in NIH3T3 cells untreated or TNFa treated at 20ng/ml for 5 minutes by cell signaling (manufacturer)<br>H2B: Acid extract from sodium butyrate treated HeLa cells by Millipore (manufacturer)<br>H2Bub11: PC-12 cell lysate by Millipore (manufacturer)<br>acetyl-Histone H3: Acid extract from sodium butyrate treated HeLa cells by Millipore (manufacturer)<br>H3: Acid extracts from colcemid treated HeLa cells by Millipore (manufacturer)<br>H3K79me2: CTH and NIH3T3 cell lines by abcam (manufacturer)<br>MLL: HeLa, HEK293T, Jurkat cell lines by bethyl laboratories (manufacturer)<br>H2Bub1: HeLa, NIH3T3, H-4-II-E, COS-7 cell lines by cell signaling (manufacturer)<br>acetyl-alpha tubulin: HeLa cell line by millipore (manufacturer)<br>vimentin: HeLa cell line by thermo fisher (manufacturer)

Eukaryotic cell lines

Policy information about cell lines and Sex and Gender in Research

Cell line source(s)	DSMZ (SEM), ATCC (NIH3T3 and REH)
Authentication	SEM - PCR for KMT2A fusion gene; NIH3T3 and REH by STR Profiling;
Mycoplasma contamination	all cell lines tested negative for mycoplasma

Commonly misidentified lines (See ICLAC register)	no misidentified cell lines were used

Animals and other research organisms

Policy information about studies involving animals; ARRIVE guidelines recommended for reporting animal research, and Sex and Gender in Research

Laboratory animals	Female NSG mice purchased from Jackson laboratories, (strain #005557) age 4 to 8 weeks. Mice were housed with a 12 hour light / 12 hour dark cycle with temperatures between 65-75°F and 40-60% humidity.
Wild animals	study did not involve wild animals
Reporting on sex	female mice were used in all experiments as they have been reported to have more efficient engraftment (Blood 2010;115 (18):3704-3070.
Field-collected samples	Field collected samples were not used.
Ethics oversight	Experiments involving mice were reviewed and approved by the Institutional Animal Care and Use Committee at St. Jude and Stanford

Note that full information on the approval of the study protocol must also be provided in the manuscript.

Clinical data

Policy information about clinical studies

All manuscripts should comply with the ICMJEguidelines for publication of clinical research and a completedCONSORT checklist must be included with all submissions.

Clinical trial registration	NA, patients not eligible for a clinical trial received previously published treatment regimens for relapsed hematologic malignancies with FDA approved chemotherapy agents on a compassionate basis.
Study protocol	Published treatment regimens are referenced in the methods
Data collection	Following IRB approval charts were reviewed over a period of two weeks and deidentified data collected on a secure encrypted hard drive.
Outcomes	Complete response: morphologic remission; Partial response: 50% or greater reduction in blasts; Stable disease: no change in blasts

ChIP-seq

Data deposition

- [x] Confirm that both raw and final processed data have been deposited in a public database such as GEO.
- [x] Confirm that you have deposited or provided access to graph files (e.g. BED files) for the called peaks.

Data access links *May remain private before publication.*	GEO repository: https://www.ncbi.nlm.nih.gov/geo, GSE152816
Files in database submission	GSM4626830 H2Bub_C32-SEM_0hr; GSM4626831 H2Bub_C53-SEM_2hr; GSM4626832 H2Bub_C54-SEM_4hr; GSM4626833 H2Bub_C55-SEM_6hr; GSM4626834 H3K79me2_C44-SEM_0hr_K79; GSM4626835 H3K79me2_C45-SEM_2hr_K79; GSM4626836 H3K79me2_C46-SEM_4hr_K79; GSM4626837 H3K79me2_C47-SEM_6hr_K79; GSM4626838 INPUT_C32-SEM_0hr_WCE; GSM4626839 INPUT_C57-SEM_2hr_WCE; GSM4626840 INPUT_C58-SEM_4hr_WCE; GSM4626841 INPUT_C59-SEM_6hr_WCE; GSM4626842 INPUT_C48-SEM_0hr_WCE_K79; GSM4626843 INPUT_C49-SEM_2hr_WCE_K79; GSM4626844 INPUT_C50-SEM_4hr_WCE_K79; GSM4626845 INPUT_C51-SEM_6hr_WCE_K79; GSM6706034 INF001D_DMSO; GSM6706035 INF001D_Bortezomib; GSM6706036 INF001R_DMSO; GSM6706037 INF001R_Bortezomib; GSM6706038 INF016D_DMSO; GSM6706039 INF016D_Bortezomib; GSM6706040 INF006D_DMSO; GSM6706041 INF006D_Bortezomib; GSM6706042 INFRF02_DMSO; GSM6706043 INFRF02_Bortezomib
Genome browser session (e.g. UCSC)	the data is release on GEO

Methodology

Replicates	ChIPseq was done once followed by a quantitative ChIPseq experiment with drosophila spike in to verify the results and trend over the time course. Data from the quantitative study are shown.
Sequencing depth	All samples had >30 million human reads
Antibodies	H3K79me2 (12.8μg/ml lysate, Abcam #ab3594), H2B-ub (10μg/ml lysate, Millipore #17-650)
Peak calling parameters	The bigwig tracks were normalized to 1M uniquely mapped spike-in reads, e.g. we doubled the track height for human reads if the

	sample had 500k uniquely mapped spike-in reads. After TMM normalization we performed empirical bayes statistics after linear fitting from voom package (R 3.23, edgeR 3.12.1, limma 3.26.9) to find differential binding sites.
Data quality	We followed ENCODE guidelines for quality control of our data, details and codes can be found in our previous publications (Genome research 22, 1813-1831, 2012; and Nature Neuroscience 22, 362-373, 2019).
Software	deeptools v3.0.2-1-ac19361, voom packge (R 3.23, edgeR 3.12.1, limma 3.26.9)

Flow Cytometry

Plots

Confirm that:

- [x] The axis labels state the marker and fluorochrome used (e.g. CD4-FITC).
- [x] The axis scales are clearly visible. Include numbers along axes only for bottom left plot of group (a 'group' is an analysis of identical markers).
- [x] All plots are contour plots with outliers or pseudocolor plots.
- [x] A numerical value for number of cells or percentage (with statistics) is provided.

Methodology

Sample preparation	Samples were washed with PBS, incubated with fluorochrome labeled antibody as recommended by the manufacturer for 15 minutes at 4C, washed twice with PBS and then resuspended in PBS at a concentration of 1 million cells per 2ml
Instrument	FACS LSR II BD (BD Biosciences)
Software	FlowJo v9
Cell population abundance	A minimum of 10,000 events are included in all analyses
Gating strategy	Cells stained with isotype controls were used to establish gates for the negative population, singlets were identified and analyzed by forward scatter height versus forward scatter area density plot for doublet exclusion. All singlets were analyzed for 7AAD and annexin V.

- [x] Tick this box to confirm that a figure exemplifying the gating strategy is provided in the Supplementary Information.
